# The Translation Initiation Factor 3f (eIF3f) Exhibits a Deubiquitinase Activity Regulating Notch Activation

**DOI:** 10.1371/journal.pbio.1000545

**Published:** 2010-11-23

**Authors:** Julien Moretti, Patricia Chastagner, Stefano Gastaldello, Sara F. Heuss, Annette M. Dirac, René Bernards, Maria G. Masucci, Alain Israël, Christel Brou

**Affiliations:** 1 Institut Pasteur, Unité de Signalisation Moléculaire et Activation Cellulaire and CNRS URA 2582, rue du Dr. Roux, Paris, France; 2 Division of Molecular Carcinogenesis and Center for Biomedical Genetics, The Netherlands Cancer Institute, Amsterdam, The Netherlands; 3 Department of Cell and Molecular Biology, Karolinska Institutet, Stockholm, Sweden; Harvard Medical School, Howard Hughes Medical Institute, United States of America

## Abstract

Activation of the mammalian Notch receptor after ligand binding relies on a succession of events including metalloprotease-cleavage, endocytosis, monoubiquitination, and eventually processing by the gamma-secretase, giving rise to a soluble, transcriptionally active molecule. The Notch1 receptor was proposed to be monoubiquitinated before its gamma-secretase cleavage; the targeted lysine has been localized to its submembrane domain. Investigating how this step might be regulated by a deubiquitinase (DUB) activity will provide new insight for understanding Notch receptor activation and downstream signaling. An immunofluorescence-based screening of an shRNA library allowed us to identify eIF3f, previously known as one of the subunits of the translation initiation factor eIF3, as a DUB targeting the activated Notch receptor. We show that eIF3f has an intrinsic DUB activity. Knocking down eIF3f leads to an accumulation of monoubiquitinated forms of activated Notch, an effect counteracted by murine WT eIF3f but not by a catalytically inactive mutant. We also show that eIF3f is recruited to activated Notch on endocytic vesicles by the putative E3 ubiquitin ligase Deltex1, which serves as a bridging factor. Finally, catalytically inactive forms of eIF3f as well as shRNAs targeting eIF3f repress Notch activation in a coculture assay, showing that eIF3f is a new positive regulator of the Notch pathway. Our results support two new and provocative conclusions: (1) The activated form of Notch needs to be deubiquitinated before being processed by the gamma-secretase activity and entering the nucleus, where it fulfills its transcriptional function. (2) The enzyme accounting for this deubiquitinase activity is eIF3f, known so far as a translation initiation factor. These data improve our knowledge of Notch signaling but also open new avenues of research on the Zomes family and the translation initiation factors.

## Abstract 2

The translation initiation factor complex eIF3f has an intrinsic deubiquitinase activity and regulates the Notch signaling pathway.

## Author Summary

The highly conserved signaling pathway involving the transmembrane receptor Notch is essential for development, and misregulation of this pathway is linked to many diseases. We previously proposed that the Notch1 receptor is monoubiquitinated during its activation. With the aim of identifying a deubiquinating enzyme that could regulate Notch activation, we demonstrated that eIF3f, known previously as part of the multiprotein translation initiation factor eIF3 complex, harbors an enzymatic activity that acts on Notch. The activated form of Notch is able to interact with eIF3f only in the presence of the E3 ubiquitin ligase Deltex, and Notch needs to be deubiquitinated before it can be cleared and its intracellular domain can enter the nucleus and fulfill its transcriptional function. Our results further decipher the molecular mechanisms of Notch signaling activation, showing that ubiquitination and deubiquitination events are required. Additionally, we show that beyond acting as a translation initiation factor, eIF3f fulfills other functions and has an intrinsic enzymatic activity.

## Introduction

Notch signaling relies on two consecutive cleavages of the receptor after binding of its ligand expressed by a neighboring cell. These two processing steps successively performed by a protease of the ADAM family and by the γ-secretase complex can occur only if the activated receptors on one side, the ligands on the other side, undergo post-translational modifications and trafficking. Some of these complex events begin to be elucidated [1]–[7]. They essentially depend on ubiquitination events affecting the ligand and/or the receptor, and probably regulating sorting and trafficking of the activated versus non-activated molecules. Eventually, after proteolytic release the intracellular portion of Notch (hereafter named NIC) enters the nucleus, where it functions as a transcriptional co-activator of Notch target genes.

In mammals, the Notch1 receptor was proposed to be monoubiquitinated before its γ-secretase cleavage; the targeted lysine has been localized to its submembrane domain [8]. Investigating how this monoubiquitination is regulated may be crucial for understanding Notch receptor activation and downstream signaling. Ubiquitination is a reversible process, and deubiquitinating enzymes (DUBs) remove the ubiquitin moieties from ubiquitinated substrates, thus allowing a tight control of these modifications [9]. A potential deubiquitination step could either affect NIC production by γ-secretase, NIC release from the endocytic vesicles, NIC entry into the nucleus, NIC interaction with its transcriptional cofactors, NIC transcriptional activity, or NIC stability. With the aim of identifying a DUB involved in Notch signaling, we established a screening strategy using shRNA vectors targeting the putative and known DUBs of the human genome [10]. Here, we report the identification of eIF3f as a DUB targeting the activated Notch receptor and positively regulating Notch signaling.

eIF3f (for eukaryotic translation initiation factor 3 subunit f) is one of the 13 subunits (named eIF3a-m) of the translation initiation factor eIF3. eIF3 stimulates many steps of the translation initiation pathway, including assembly of the eIF2-GTP/met-tRNA complex to the 40S ribosome to form the 43S preinitiation complex (PIC), mRNA recruitment to the 43S PIC complex, impairment of the 40S ribosome to join the 60S prematurely, and scanning the mRNA for AUG recognition [11]. eIF3 has no known enzymatic activity so far, but it has an intriguing degree of homology with two other complexes whose functions appear unrelated: the COP9 signalosome and the 19S proteasome lid. All three complexes, forming the Zomes family, consist of subunits with either PCI (Proteasome-COP9 signalosome-initiation factor 3 domain) or MPN (for MPR1-PAD1-N-terminal domain) signature domains and share a common 6PCI +2 MPN domain stoechiometry. The mammalian eIF3 also has an additional five non-PCI-MPN subunits [12]. The MPN domain of CSN5 (COP9 Signalosome subunit 5) harbors a metalloprotease motif referred to as the Jab/MPN domain-associated metallopeptidase (JAMM) motif and regulates the activity of E3 ubiquitin ligases by deneddylation of the cullin component. On the other hand, a JAMM-containing subunit associated with the 19S proteasome lid (Rpn11, [13]) also harbors DUB activity, accounting for substrates deubiquitination before they enter the proteasome channel. Interestingly, eIF3f contains a JAMM domain, making it a putative DUB [14],[15]. We show here that it harbors a DUB activity acting on Notch signaling.

## Results

### Identification of eIF3f

**Figure 1:**
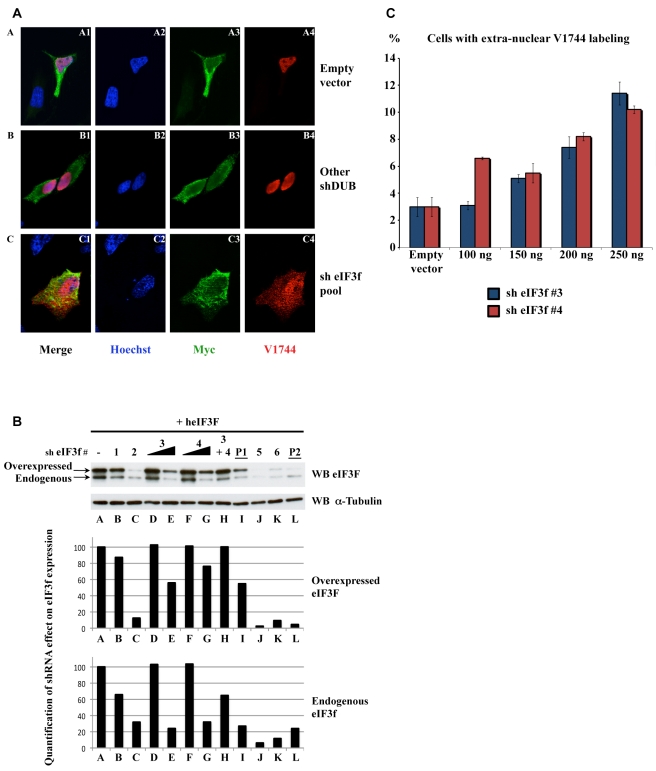
Identification of eIF3f. (A) Immunofluorescence screen. U2OS cells were transfected with a ΔE expressing vector together with each pool of shRNAs targeting the human DUBs. Notch products were detected with anti-myc antibody, or with V1744, to specifically detect NIC (secondary antibodies were coupled with Alexa-488 and Alexa-555, respectively). Nuclei were stained with Hoechst. (B) Western blot analysis of endogenous eIF3f and transfected HA-tagged eIF3f in the presence of the pooled (P1 and P2) or the individual shRNA vectors (#1 to #4 from P1, #5 and #6 from P2) targeting eIF3f. α-tubulin loading control is shown. Under the lanes is shown the quantification of the eIF3f bands (overexpressed or endogenous as indicated), performed with the Quantity One program (Biorad). (C) Quantification of the effect of two shRNA vectors targeting eIF3f (#3 and #4, respectively, in blue and red) on the extranuclear localization of NIC. Transfection and immunofluorescence were performed as in (A). 400 Notch-positive cells were counted for each point and the percentage of cells showing extranuclear V1744 labeling is shown. Error bars represent the SEM of duplicate experiments.

In order to identify DUBs involved in the Notch signaling pathway, we set up an immunofluorescence screen. We used Notch ΔE [16], a mutant form of Notch deleted of most of its extracellular domain. ΔE mimics the ADAM-cleavage product and therefore represents a constitutively active form, which is monoubiquitinated and endocytosed [8] before being cleaved by γ-secretase to liberate NIC [17]. We made use of the V1744 antibody to monitor the production and the localization of NIC, whereas anti-myc antibody detected all Notch products. U2OS cells were co-transfected with vectors encoding ΔE and with each individual pool of a shRNA library targeting the 91 known or putative DUBs encoded by the human genome (called shDUB library; see Table 1) [10]. With ΔE alone (Figure 1A, Panel A), we observed a membrane and endomembrane-localized myc labeling corresponding to the unprocessed Notch ΔE, but also a nuclear myc labeling (A3) corresponding to NIC, co-stained with the V1744 antibody (A4). The same pattern was observed with almost every shRNA pool of the library (exemplified in Panels B1–4), whereas four pools abolished ΔE expression (those targeting eIF3h, PRP8 and USP54, and Pool 2, see below). However, with an shRNA pool targeting eIF3f (panels C), we observed a partial extra-nuclear V1744 labeling (C4), as well as an increase in the average proportion of extranuclear/nuclear myc staining (see Figure S1, Dose 1). This suggests that NIC production and localization was affected when eIF3f was knocked down.

The shRNA library actually contained two pools (each containing four shRNA-encoding plasmids) targeting eIF3f: Pool 1, whose effects are shown in Figure 1A, and Pool 2, in the presence of which no ΔE-positive signal could be detected by immunofluorescence (unpublished data). We isolated the eight shRNAs from these pools (six different shRNAs in total) and tested them individually for their efficiency in knocking down eIF3f: Pool 1 (P1) contains shRNA #1 to #4, Pool 2 (P2) contains shRNA #1, #2, #5, and #6. HEK293T cells were transfected with each of these shRNAs and the levels of endogenous or overexpressed human HA-tagged eIF3f were analyzed by Western blotting. Anti-α-Tubulin was used as a loading control (Figure 1B, bottom panel), since this protein is very stable and reflects the total protein content in transfected and non-transfected cells. shRNAs #1 and #2 significantly affected the level of endogenous and transfected eIF3f (Figure 1B, Lanes B and C and quantification under the lanes), similar to the Pool 1 (P1, Lane I). The additional shRNAs from Pool 2 (#5 and #6 in Lanes J and K) and Pool 2 itself (P2, Lane L) almost completely abolished eIF3f expression in this assay. The effect of each of these shRNAs on eIF3f protein level was correlated with its ability to inhibit ΔE expression in immunofluorescence experiments and probably reflects eIF3f requirement for translation (Figure S1). Indeed shRNAs #3 and #4, which exhibited a minor effect on the expression of the eIF3f protein (Figure 1B, Lanes D–G), were the most efficient in inducing extranuclear V1744 labeling in a dose-dependent manner (Figure 1C). Nevertheless, further increasing the dose of these two shRNAs separately or co-transfecting them resulted in an effect on eIF3f expression (Figure 1B, Lanes E, G, and H) and an extinction of the ΔE signal in immunofluorescence (Figure S1 and unpublished data). Since the shRNAs that affect Notch signaling target different sequences of eI3Ff, and since this effect is complemented by transfection of wt eIF3f (see below), we can exclude an off-target effect of these shRNAs. Taken together, these results suggest that a specific but mild knockdown of eIF3f is responsible for the altered Notch localization observed in Figure 1.

### eIF3f Is Involved in the Deubiquitination of Activated Notch

**Figure 2:**
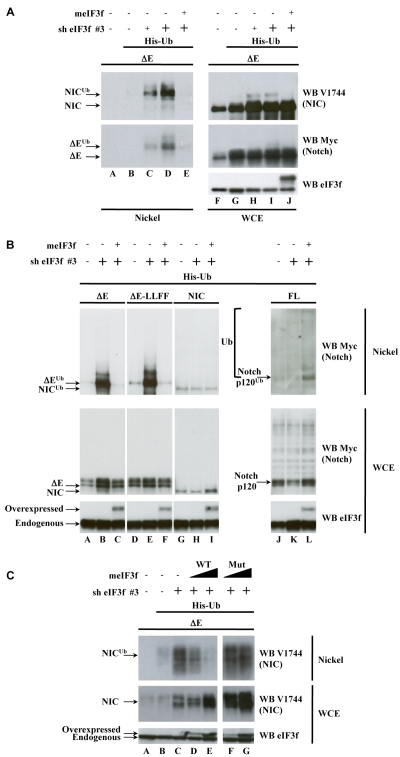
eIF3f is involved in the deubiquitination of activated Notch. HEK293T cells were transfected with: (A) ΔE, His-ubiquitin, two increasing doses of eIF3f shRNA #3 (indicated by + and +, respectively), and meIF3f as indicated; (B) ΔE, ΔE-LLFF, NIC or Notch full length (FL), His-ubiquitin, the larger dose of eIF3f shRNA used in (A) (+), and meIF3f as indicated; (C) ΔE, His-ubiquitin, shRNA #3, and two doses of meIF3f WT or Mut as indicated by a filled triangle. In all panels, cells were lysed under denaturing conditions and subjected to Nickel purification. Ni-purified material or WCE (5% of the total lysates) were resolved on SDS-PAGE and analyzed by Western blot using the antibodies indicated on the right of the panels. Notch p120 designates the membrane-anchored form of non-activated full-length Notch, resulting from furin cleavage. Overexpressed meIF3f has a higher apparent molecular weight than the endogenous human eIF3f because of its HA tags. White lines indicate that intervening lanes have been spliced out.

We next tested the effect of eIF3f on the ubiquitination of activated Notch. HEK293T cells were transfected with vectors encoding ΔE and a 6xHis-tagged Ubiquitin. We added increasing amounts of shRNA #3, without reaching the doses used in Figure 1B, Lane E, in the presence or not of a murine form of eIF3f (meIF3f). The murine eIF3f cDNA exhibits a three base pair change in the sequence targeted by shRNA #3 and is therefore refractory to its effect. Proteins were extracted in denaturing conditions and ubiquitinated proteins were purified on Nickel-charged beads. Finally, whole cell extracts and ubiquitinated products were analyzed by Western blot to quantify the levels of ubiquitinated Notch (Figure 2A). Transfection of shRNA #3 led to a dose-dependent accumulation of monoubiquitinated ΔE (ΔE^Ub^) and of monoubiquitinated NIC (NIC^Ub^) (Lanes C, D compared to B), whereas the levels of Notch in the extracts remained stable (Lanes G to J). The same effect was observed using shRNA #4 (unpublished data). Interestingly, overexpression of meIF3f abolished the effect of shRNA #3 and accumulation of ubiquitinated Notch could no longer be seen (compare Lanes E to D). The ubiquitination levels of other forms of Notch were also analyzed in the presence or absence of shRNA #3 (Figure 2B): the membrane-anchored ΔE-LLFF (Lanes D–F), which undergoes the same processes as ΔE (i.e., monoubiquitination and endocytosis) but which is mutated in the γ-secretase cleavage site and consequently does not generate NIC [18]; the nuclear Notch (NIC, Lanes G–I) and the non-activated full-length Notch (FL, Lanes J–L). The only forms whose ubiquitination was affected by shRNA #3 and/or meIF3f were those corresponding to activated and still membrane-anchored forms of Notch, namely ΔE and ΔE-LLFF. We also tested as controls NDFIP2 (a transmembrane protein located in the endosomal compartment [19]) and Deltex1 (an E3 ubiquitin ligase genetically identified as involved in Notch signaling). These two proteins showed no change in ubiquitination in the presence of shRNA #3 or overexpressed meIF3f (Figure S2 and unpublished data). These results suggest that eIF3f is specifically involved in an ubiquitination/deubiquitination process targeting activated Notch.

The eIF3f protein contains an MPN domain, also found in some DUBs of the JAMM family, such as AMSH, Rpn11, and CSN5 [20]–[23]. However, the position of the amino acids constituting the catalytic site of these DUBs is not strictly conserved in eIF3f, although histidines and acidic residues can still be found and can possibly form a signature of metalloprotease (HEX_2_HX_2_GX_2_H). We mutated six amino acids of eIF3f, including two histidines and two acidic residues of the putative catalytic site. We then tested in parallel WT meIF3f and this putative catalytic mutant (meIF3f Mut) (Figure 2C). Whereas WT meIF3f was able to prevent shRNA #3–induced NIC^Ub^ accumulation in a dose-dependent manner (Lanes D, E compared to C), meIF3f Mut was not, although it was expressed at similar levels (Lanes D to G). This shows that meIF3f indeed complements the effect of shRNA #3 and that an intact MPN domain of eIF3f is necessary for its effect on monoubiquitination of activated Notch. Taken together, these results strongly suggest that eIF3f could act as a DUB targeting activated Notch.

### eIF3f Harbors a DUB Activity

**Figure 3:**
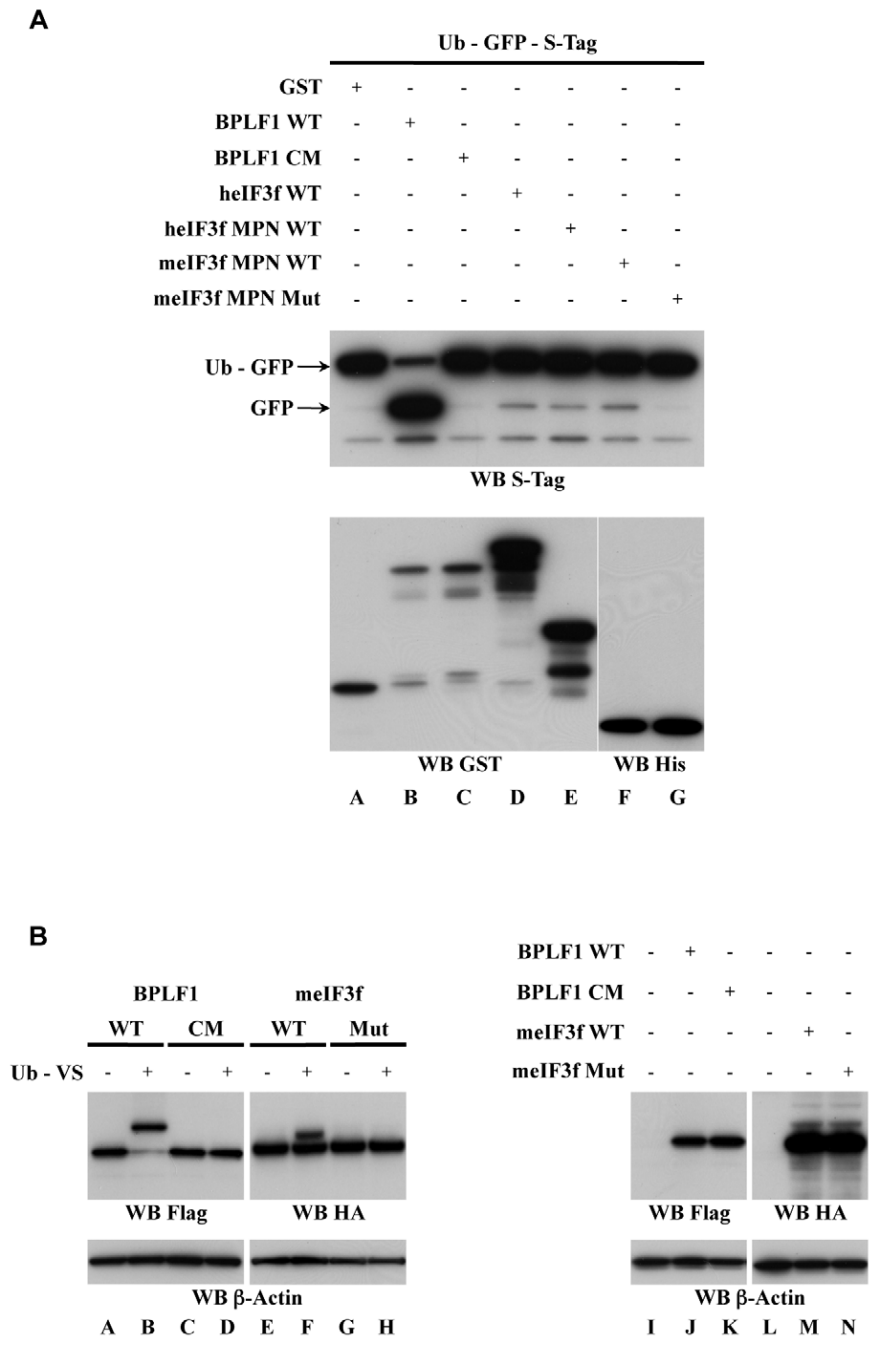
eiF3f DUB activity. (A) eIF3f exhibits a DUB activity in a bacterial Ub-GFP assay. Bl21 bacteria were transformed with Ub-GFP-S-Tag plasmid and either GST-fusions or His-fusions as indicated. After protein expression induction, they were lysed by sonication. WCE were analyzed by Western blot using the antibodies indicated under each panel to visualize GFP release from Ub-GFP (WB S-Tag) and to control protein expression (WB GST and His). The GFP product resulting from the DUB activity and the Ub-GFP substrate are indicated by the arrows. (B) eIF3f binds ubiquitin in an eukaryotic Ub-VS assay. HeLa cells were transfected with expression vectors encoding BPLF1 WT or CM (Lanes A–D, J, K), or meIF3f WT or Mut (Lanes E–H, M, N) as indicated. 36 h after transfection, 10 µg of WCE were incubated (or not) with Ub-VS probe (Lanes A to H). Samples were finally analyzed by Western blot as indicated under the panels to monitor Ub-VS binding (Lanes A to H) and to check protein expression (Lanes I to N). β-Actin was used as a loading control. The apparent molecular weights of BPLF1 and meIF3f are 32 kDa and 50 kDa, respectively.

In order to test whether eIF3f exhibits a deubiquitinase activity, we first used a functional in bacteria assay using GFP fused to ubiquitin (Ub-GFP) [24]. The peptide bond between ubiquitin and GFP can be cleaved by ubiquitin- or UbL-deconjugase activities, which are absent from the bacterial genome. Bl21 bacteria were transfected with plasmids encoding GST alone, GST-fused to human WT eIF3f or WT MPN domain, or His-tagged murine eIF3f WT or mutant MPN domain (Mut), together with a vector encoding Ub-GFP fused to an S-Tag at its C-terminus (Ub-GFP-S-Tag) (Figure 3A). As control DUB, we used BPLF1 WT (an EBV DUB of the cysteine-protease family [24]) and its catalytically inactive form BPLF1 CM. After induction of protein expression, the bacteria were lysed and cleavage of Ub-GFP was assessed in Western blot by the appearance of free GFP-S-Tag using anti-S-Tag antibody (Figure 3A, upper panel). Anti-GST or anti-His antibodies were used to verify protein expression (Figure 3A, bottom panel). As expected, we observed released GFP with BPLF1 WT but not its CM mutant (compare Lanes A–C). Interestingly, we also detected protease activity with heIF3f WT, heIF3f MPN WT, and meIF3f MPN WT (Lanes D, E, and F, respectively) but not with the mutant meIF3f MPN Mut (Lane G).

To further investigate whether eIF3f could act as an ubiquitin-specific protease, we performed an in vitro assay using Ubiquitin Vinyl Sulfone (Ub-VS), a functional probe that covalently binds the active site of DUBs and consequently inhibits DUB catalytic activity (Figure 3B). HeLa cells were transfected with vectors encoding HA-tagged meIF3f, either WT or Mut. Flag-tagged BPLF1 WT and CM were used as controls. A portion of the corresponding whole cell extracts was incubated in vitro with Ub-VS. Total extracts incubated or not with Ub-VS were finally analyzed by Western blot using anti-HA and anti-Flag antibodies to monitor the covalent binding of Ub-VS to the DUBs (Figure 3B, Lanes A to H) and to check protein expression (Lanes I to N), respectively. We observed a partial upshift corresponding to the size of the Ub-VS probe with meIF3f WT (Lane F compared to E) and with BPLF1 WT (Lane B compared to A), indicating that both are able to bind Ub-VS. In contrast, we did not observe any shift with meIF3f Mut (Lane H compared to G) nor BPLF1 CM (Lane D compared to C), showing that they are indeed unable to bind Ub-VS. We obtained the same results when the DUBs were expressed in bacteria (unpublished data).

Taken together, these results show that eIF3f exhibits a deubiquitinase activity, carried by its MPN domain. Moreover it confirms that the mutant we generated (Mut) by replacing six amino acids of the metalloprotease-like sequence of eIF3f is a catalytically inactive form of eIF3f.

### Deltex1 Is an Intermediate between eIF3f and Notch

**Figure 4:**
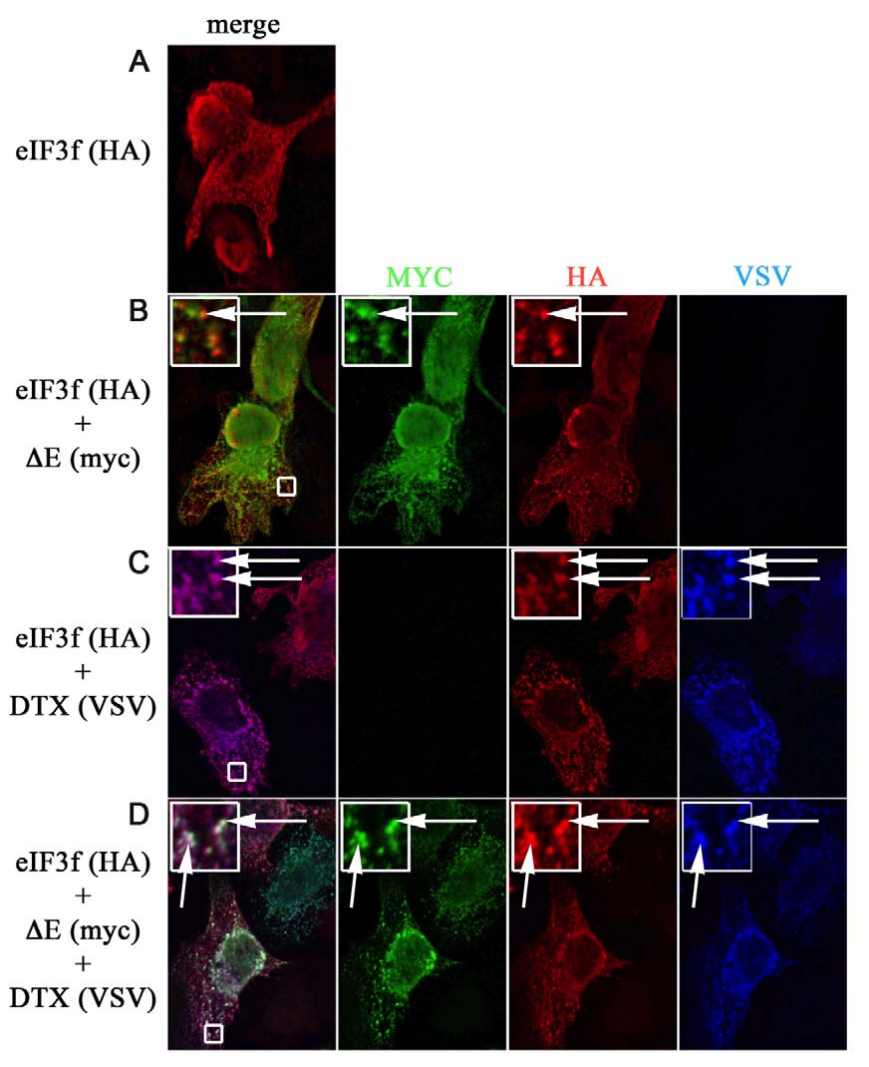
Colocalization of eIF3f, DTX, and ΔE. U2OS cells were transfected with expression vectors as indicated on the left. eIF3f, DTX, and ΔE were detected using mouse anti-HA, rabbit anti-VSV, and goat anti-myc, respectively (with CY3-coupled, Alexa 488-coupled, and Alexa 647-coupled secondary antibodies, respectively). Insets represent enlargements (4-fold) of the boxed region, arrows indicate colocalizations, and left panels are merges of the three adjacent panels.

In order to identify the site of action and the target of eIF3f in the Notch activation cascade, we first overexpressed ΔE and murine eIF3f in U2OS cells. We observed by immunofluorescence a low frequency of vesicular colocalization of the two proteins (Figure 4, Panels A and B). As eIF3f might require a cofactor or target an intermediate protein to regulate Notch ubiquitination, we tested several components of the Notch signaling pathway that could be associated with trafficking. Some DUBs are known to be recruited to their substrate indirectly via an E3 ubiquitin ligase [25],[26], so we particularly focused on the E3 ubiquitin ligases of the Notch pathway known to be associated with the endocytic machinery: Itch/AIP4 and Deltex1 (hereafter designated as DTX) ([27] and therein). In contrast to Itch/AIP4, DTX significantly colocalized with eIF3f when both proteins were coexpressed (Figure 4, Panel C). In addition, the presence of DTX strikingly increased the colocalization of ΔE and eIF3f, the three proteins being associated to the same vesicles (Figure 4, Panel D). These results suggest that DTX could recruit eIF3f to activated Notch.

**Figure 5:**
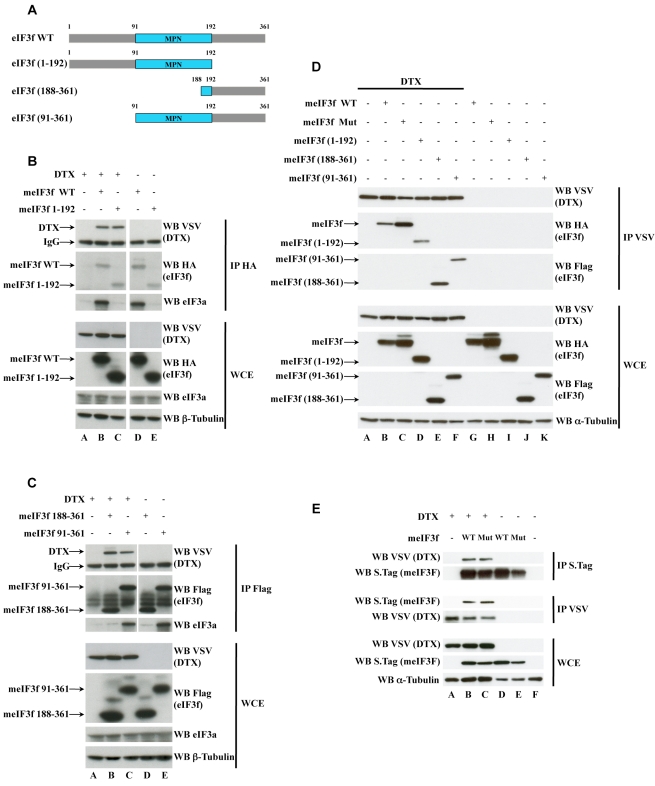
Interactions of eIF3f and DTX. (A) Schematic representation of murine eIF3f deletion mutants. The conserved MPN domain is depicted in blue; amino acid coordinates are indicated. (B) HEK293T cells were cotransfected with vectors encoding HA-tagged eIF3f WT or (1–192), and VSV-tagged DTX. Whole cell extracts (WCE, 5% of the lysates) were either directly blotted with antibodies indicated on the right of the lanes or immunoprecipitated first with anti-HA antibody. β-tubulin was used as a loading control. (C) HEK293T cells were cotransfected with vectors encoding Flag-tagged eIF3f (188–361) or (91–361), and VSV-tagged DTX. WCE (5%) were either directly blotted with antibodies indicated on the right of the lanes or immunoprecipitated first with anti-Flag antibody. β-tubulin was used as a loading control. (D) HEK293T cells were cotransfected with vectors encoding the various eIF3f mutants and VSV-DTX. WCE were immunoprecipitated with anti-VSV antibody, and the precipitates were eluted with VSV peptide before being loaded on SDS gels. The eluted material and the WCE (5% of the lysates) were analyzed by Western blotting with antibodies indicated on the right of the lanes. α-tubulin was used as a loading control. (E) Stable cell lines derived form MEFs by retroviral transduction of VSV-DTX or S-tagged eIF3f (either WT or active site mutant) were lysed and subjected to parallel immunoprecipitations with VSV and S-tag antibodies as indicated. The VSV- or Laemmli-eluted material (for VSV and S-tag IPs, respectively) and the WCE (5% of the lysates) were analyzed by Western blotting with antibodies indicated on the right of the lanes. α-tubulin was used as a loading control. White lines indicate that intervening lanes have been spliced out.

To verify this hypothesis, we performed co-immunoprecipitation experiments in HEK293T cells transfected with vectors encoding VSV-tagged DTX, Flag-Itch/AIP4, and HA- or Flag-tagged forms of eIF3f (schematized in Figure 5A). We pulled down eIF3f and analyzed the whole cell extracts and the immunoprecipitates by Western blot (Figure 5B, 5C and Figure S3). DTX co-immunoprecipitated with eIF3f WT and (1–192), but also with eIF3f (188–361) and (91–361) (compare Lanes B, C to D, E in Figure 5B and 5C). No co-immunoprecipitation was detected with Itch/AIP4 (Figure S3). As a control, the endogenous eIF3a, another subunit of the eIF3 complex, only co-immunoprecipitated with eIF3f WT and (91–361) (Panels B, C). This observation suggests that DTX is able to physically interact with eIF3f and that the domain(s) necessary for this interaction is different from the domain necessary for eIF3f to incorporate the eIF3 complex. We then performed the reverse experiment by pulling down VSV-tagged DTX and could detect the various forms of eIF3f coimmunoprecipitating with DTX (Figure 5D): WT or Mut eIF3f (Lanes B, C) as well as the deletion mutants (Lanes D–F). To confirm these results under conditions where the proteins are not overexpressed, we established by retroviral transduction murine cell lines expressing low amounts of VSV-DTX and S-tagged forms of WT eIF3f or of a mutant of the active site (HDI to AAA). eIF3f or DTX was immunoprecipitated first and their association was confirmed in both cases (Figure 5E).

**Figure 6:**
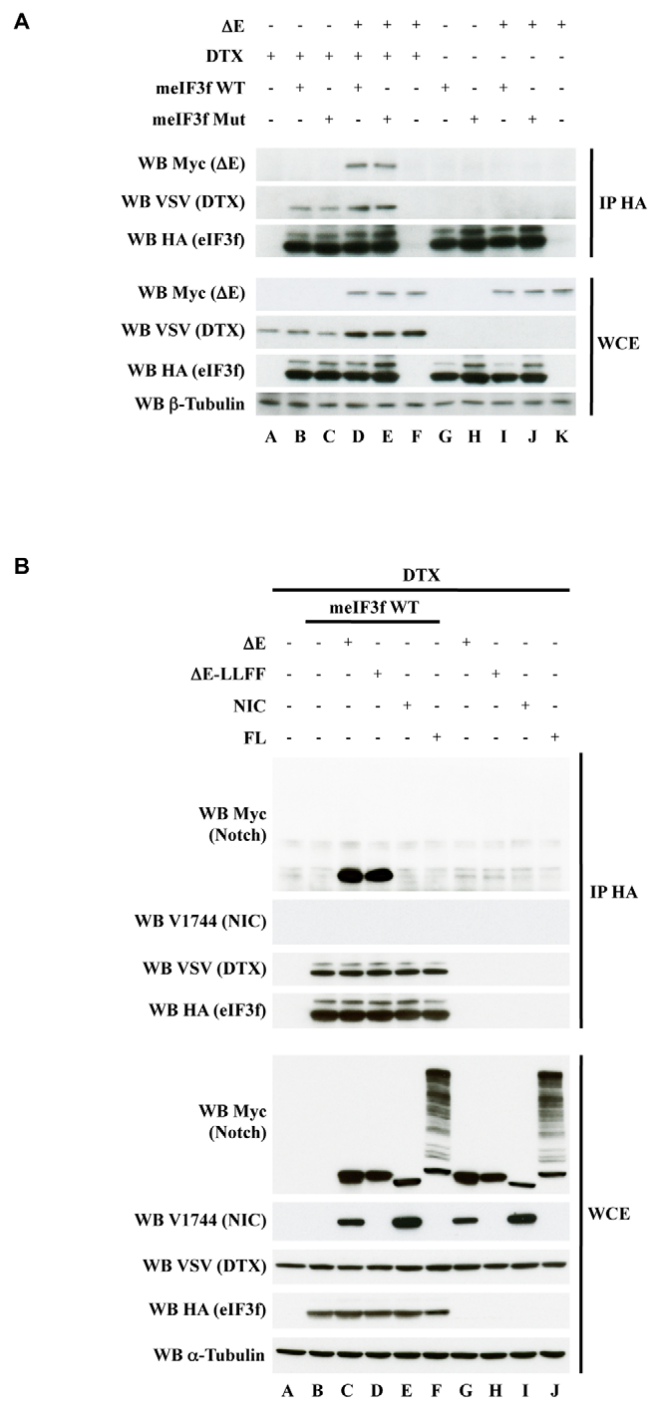
Interaction of eIF3f and activated Notch in the presence of DTX. HEK293T cells were cotransfected with vectors encoding HA-tagged forms of eIF3f (either WT or Mut in Panel A), VSV-DTX, and myc-tagged Notch constructs (ΔE, ΔE-LLFF, NIC, or FL in Panel B) as indicated above the lanes. Immunoprecipitations were performed with anti-HA antibody. In both panels, immunoprecipitates and WCE (10% of the lysates) were analyzed by Western blot as indicated. α- or β-tubulin were used as loading controls.

We then performed co-immunoprecipitations in HEK293T in the presence of Notch ΔE (Figure 6A). While DTX co-immunoprecipitated with WT eIF3f but also with meIF3f Mut (Figure 6A, Lanes B–E), ΔE did not (Lanes I and J), unless DTX was cotransfected (Lanes D and E). This strongly suggests that a tripartite interaction occurs between activated Notch, DTX, and eIF3f, DTX being required for the Notch-eIF3f interaction. In addition, we verified that neither DTX nor ΔE could be co-immunoprecipitated with eIF3f from cell extracts that were transfected separately and mixed (unpublished data). We then repeated these experiments using other forms of Notch: ΔE-LLFF, NIC, or the non-activated full-length Notch (FL) (Figure 6B). Only two forms could co-immunoprecipitate with eIF3f in the presence of DTX: ΔE and ΔE-LLFF (Lanes C and D). No signal was detected with FL and NIC (Lanes E and F) even with a longer exposure. It is of note that NIC produced from ΔE (Lanes C, G) and detected by the V1744 antibody was not co-immunoprecipitated with eIF3f either. These results show that the tripartite interaction between Notch, DTX, and eIF3f occurs preferentially with activated, membrane-associated, but γ-secretase unprocessed, forms of Notch.

Our results (Figures 2 and 6) suggest that the target of eIF3f is preferentially a γ-secretase unprocessed form of activated Notch. The fact that we could detect ubiquitinated NIC in the presence of shRNAs #3 and #4 (Figure 2A) might thus appear paradoxical, however it might be the consequence of γ-secretase cleavage of non-deubiquitinated Notch ΔE. We also observed that shRNAs #3 and #4 led to a partially extranuclear localization of NIC (Figure 1C). This suggests that NIC carrying residual monoubiquitination could be impaired in nuclear translocation. In order to test this possibility, we tried to mimic NIC^Ub^ by attaching an ubiquitin to the N-terminus of NIC (UBIC construct). Given that ΔE, and consequently any putative non-deubiquitinated NIC, has been shown to be monoubiquitinated on K1749, we used a K1749R mutant of NIC (NIC(KR)) on which no ubiquitin can be conjugated. In addition, the attached ubiquitin was mutated on the internal Lysine residues 29 and 48 to impair polyubiquitination and also on the 2 C-terminal glycines to prevent proteolysis by a DUB enzyme [28]. U2OS cells were transfected with NIC, NIC(KR), or the new construct UBIC. We observed by immunofluorescence that NIC or NIC(KR) were mostly nuclear (96% in average), whereas UBIC was partially retained in the cytoplasm (18% see Figure S4, Panel A). In addition, we monitored the transcriptional activity of these three forms by cotransfecting U2OS cells with increasing doses of expression vectors together with a Notch-reporter gene (CSL-luciferase, [29],[30]) and an internal control reporter (pRL-TK). As shown in Figure S4, Panel B, UBIC is significantly less active than NIC or NIC(KR), although it is expressed at a comparable level (see Western blot in Figure S4, Panel B bottom). One possibility to explain the localization and the drop in transcriptional activity of UBIC is that the ubiquitin moiety partially prevents access to the NLS of NIC.

### eIF3f Is a Positive Regulator of Notch Signaling

**Figure 7:**
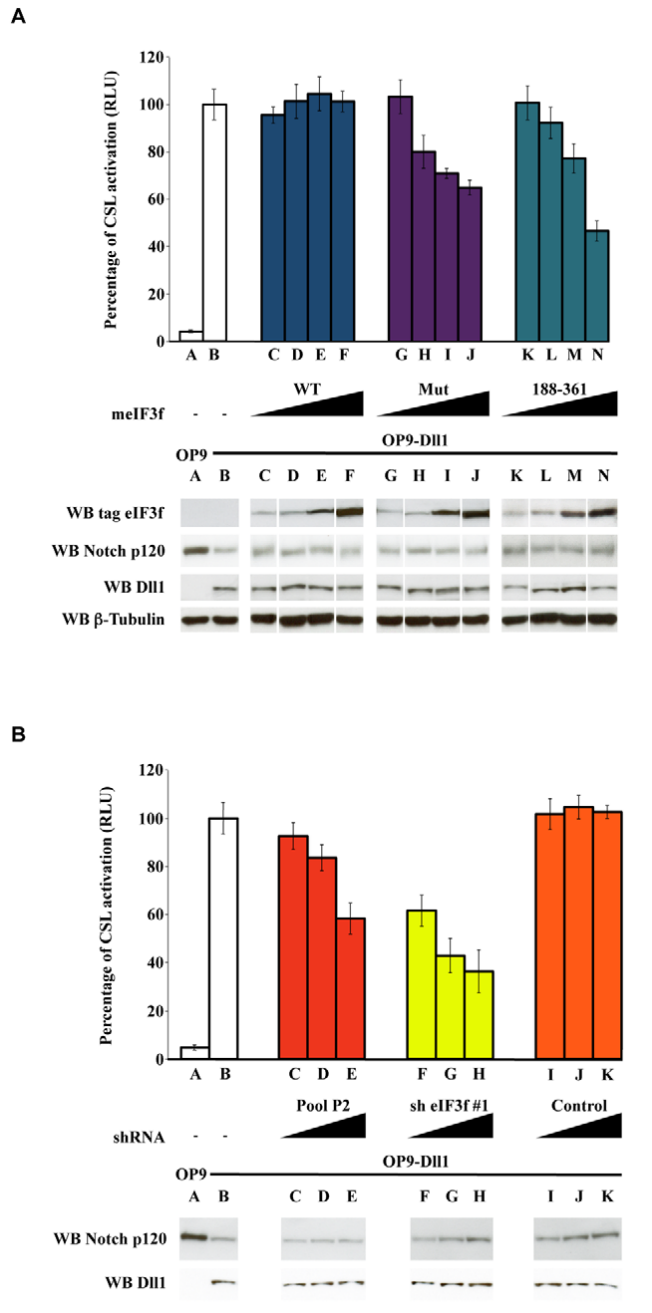
Notch-Delta coculture assay. Modulation of the Notch reporter activity (CSL-luciferase) in Notch1-expressing cells cocultured with Dll1-expressing cells (Lanes B–N) or control cells (Lane A). The Notch-expressing cells were first transfected with both CSL-firefly luciferase (Notch reporter) and TK-renilla luciferase (internal control) 24 h before coculture. The CSL-reporter activation corresponds to the ratio between Firefly and Renilla luciferase activities. The relative luciferase activity in the presence of Dll1-expressing cells (Lane B) was defined as 100%. Error bars represent the SEM of triplicate experiments. (A) Effects of increasing doses of expression vectors encoding eIF3f WT (Lanes C–F), Mut (G–J), or (188–361) (K–N) in reporter cells are shown as indicated. Expression levels of eIF3f, Notch, and Dll1 were confirmed by Western blot analysis, using β-tubulin as a loading control. White lines indicate that intervening lanes have been spliced out. (B) shRNAs targeting eIF3f decrease Notch signaling. Increasing doses of eIF3f shRNA P2 (C–E), shRNA #1 (F–H), or a control pool targeting AMSH (I–K) were transfected into the Notch-expressing cells as above. Bottom shows the Western blot analysis of a representative experiment.

In order to test whether eIF3f could act on Notch signaling under more physiological conditions, we performed a coculture assay using a CSL reporter strategy. U2OS cells stably expressing Notch FL were transfected with a CSL-Luciferase Notch reporter and increasing doses of meIF3f WT, meIF3f Mut, or meIF3f (188–361). pRL-TK vector, encoding Renilla luciferase under the control of the Notch-insensitive thymidine kinase promoter, was also cotransfected as an internal control. These cells were then cocultured with OP9 cells stably expressing or not the Notch ligand Delta-like1 (Dll1) [31], and relative luciferase activity was finally determined by normalizing CSL-firefly luciferase with renilla luciferase. In parallel cell extracts from the same transfections were analyzed by Western blot (Figure 7A, bottom). In the presence of Dll1, the relative luciferase activity of the CSL reporter gene increased 20-fold (Figure 7A, Lanes A, B), showing that Notch was indeed activated by Dll1. While the presence of meIF3F WT did not modify Dll1-dependent Notch activation (Lanes C to F), meIF3F Mut and meIF3f (188–361) repressed it in a dose-dependent manner, respectively, reaching 35% and 54% of reduction (Lanes G–J and K–N, respectively). Thus, meIF3f Mut and meIF3f (188–361) inhibit Notch transcriptional activity and behave as dominant-negative forms of eIF3f, in accordance with the fact that both lack an intact catalytic site but are able to bind DTX and Notch. We also tested the effect of shRNAs targeting endogenous eIF3f in this assay. As represented in Figure 7B, Dll1-induced Notch stimulation was inhibited in the presence of increasing doses of either P2 (Lanes C–E) or shRNA #1 (Lanes F–H), although Notch1 overall level remained constant (see bottom of Figure 7B). In contrast, a shRNA pool targeting AMSH, another DUB of the JAMM family, had no effect on Notch activation (Lanes I–K). Furthermore, the same shRNAs have no effect on NIC-mediated transcriptional activation (Figure S5), excluding any effect of the shRNAs on Notch-associated transcription factors. Therefore, inhibition of eIF3f DUB activity impairs the production of a transcriptionally active NIC in a dose-dependent manner.

All together, our results indicate that eIF3f, after being recruited to vesicular Notch via DTX, could act as a DUB on a monoubiquitinated but not γ-secretase processed form of Notch, thus positively modulating Notch signaling activity.

## Discussion

### A DUB Acting during Notch Activation

With the aim of identifying a DUB that could regulate Notch activation, we reached two new and provocative conclusions: the activated form of Notch needs to be deubiquitinated to enter the nucleus and fulfill its transcriptional function, and the DUB accounting for this activity is eIF3f, known so far as a translation initiation factor. When reconstituting a Notch activation system by co-culturing Notch1 receptor- and Dll1 ligand-expressing cells, we observed that Notch-dependent transcriptional activation of a reporter gene was specifically affected when expressing mutant forms of eIF3f where the active site was mutated or when eIF3f was partially knocked down. Therefore eIF3f acts as a positive regulator of Notch signaling, independently of its function in translation (see below). We have also demonstrated that a monoubiquitinated form of Notch ΔE was stabilized when eIF3f was slightly knocked down. This effect was accompanied by the appearance of a mono-ubiquitinated form of NIC, which is probably excluded from the nucleus. Therefore we conclude that Notch deubiquitination is necessary for its full activity in the nucleus.

We have not formally identified the form of Notch that is the substrate of the DUB activity. We cannot exclude that it is a monoubiquitinated non-nuclear NIC, resulting from γ-secretase cleavage of activated Notch. Such a form has never been detected before, in contrast to the nuclear polyubiquitinated NIC, which appears subsequently under the action of the E3 ubiquitin ligase Sel10 [32],[33]. An artificial construct mimicking to a certain extent the monoubiquitinated NIC (UBIC) was partially retained in the cytoplasm and as a consequence was transcriptionally less active than NIC. This suggests that the ubiquitin moiety could mask the proximal NLS [34] and impair interaction with importins. Nevertheless three observations argue against the possibility of NIC being the natural substrate of eIF3f: first, NIC was not obviously associated with endosomes when it was excluded from the nucleus in the presence of eIF3f shRNA. As eIF3f colocalizes with Notch to endosomal structures, its target should be retained to these structures (Figure 4). Second, NIC produced from ΔE was not detected as coimmunoprecipitating neither with eIF3f nor with its mutant in the presence of DTX, which would have probably been the case if it were the DUB substrate (Figure 6). And third, the effect of shRNAs targeting eIF3f were only detected on activated but still membrane-anchored forms of Notch, including one unable to produce NIC (ΔE-LLFF, see Figure 2). Therefore we favor the hypothesis that the eIF3f substrate is the activated, monoubiquitinated but still membrane-anchored Notch (mimicked by ΔE in transfection experiments, see [8]). The presence of monoubiquitinated NIC when eIF3f activity is impaired would be due to a partial activity of γ-secretase on non-deubiquitinated ΔE.

Finally, our results show that eIF3f is recruited to Deltex1 and Notch ΔE-containing vesicles in transfected cells. They can thus form a tripartite complex where DTX serves as a bridging factor between Notch and eIF3f. Deltex1 belongs to the RING family of E3 ubiquitin ligase, however its target in Notch signaling remains to be determined [35]. It localizes to the endocytic pathway [27], interacts with Notch, and thus could serve as a scaffolding protein enabling Notch trafficking and modifications, and eventually Notch signaling.

### eIF3f Is a DUB

Our results suggest that eIF3f, known so far as a component of a translation initiation factor complex, is itself able to act as a DUB during Notch activation. In contrast to a mutant of the active site, a WT form of eIF3f is able to complement the inhibition of activity mediated by eIF3f shRNAs and to restore deubiquitination of Notch ΔE. However we cannot completely rule out the possibility that another DUB, associated with eIF3f, could account for Notch deubiquitination. This is, for example, the case for CSN5, which, in addition to its own isopeptidase activity, is associated with USP15, both being required for proper processing of polyubiquitinated substrates bound to p97/VCP [36]–[38]. Such a putative eIF3f-associated DUB, the knockdown of which would not affect protein translation, should have been identified during the screen. Moreover, eIF3h, the other MPN-containing subunit of eIF3 associated with eIF3f [12], has neither histidines nor acidic residues that could confer a DUB activity. The fact that eIF3f was the only DUB identified argues against the possibility of an associated DUB. eIF3f was not identified in other screens, in particular in one recently performed in Drosophila [39], probably because a strong extinction of this translation factor had a more severe and broad phenotype than a Notch phenotype, even on external sensory organ development. We actually were able to pick up this factor thanks to the presence of relatively inefficient shRNAs in the library.

The human genome encodes 14 JAMM proteins, seven of which have a complete set of the conserved residues for Zn2+ coordination [40]. Among them, six (AMSH, AMSH-LP, BRCC36, Rpn11, MYSM1, and CSN5) have been reported to have isopeptidase activity on ubiquitin or ubiquitin-like proteins. It is of note that the JAMM sequence of eIF3f cannot be aligned with those of the proteins constituting the MPN+ group (Rpn11, Csn5, AMSH; see [15]). Nevertheless the four polar and the additional glutamate residue in a more N-terminal region of the domain are still present in eIF3f, although not arranged in the MPN+-defined pattern, and the mutation of some of these amino acids affects eIF3f enzymatic activity and function in Notch signaling. It must be noted that the MPN+ consensus domain has been generated using a small number of proteins and that divergent members may well exist, including eIF3f.

Using an in bacteria assay, we have demonstrated that full-length eIF3f or its isolated MPN domain exhibit DUB activity and that the active site indeed involves the amino acids that we had targeted in our inactive mutant. The relatively low activity that we detected in this assay, as compared to the BPLF1 control, might be due to the specificity of this DUB, which is limited by the nature of the P'1 and P'2 residues in the substrate sequence, thus preventing cleavage of non-specific substrates. However we have confirmed by Ub-VS fixation that WT but not mutant eIF3f exhibits DUB activity in mammalian cells. Among the members of the JAMM family, AMSH and AMSH-like seem to be specific for Lys63-linked polyubiquitin chains [41], while MYSM1 is specific for monoubiquitinated H2A [42]. Sato et al. [43] suggested that the specificity of AMSH family members for Lys63-linked polyubiquitin chains is primarily due to their interaction with the proximal ubiquitin, involving a single domain containing two characteristic insertions that are not conserved in eIF3f. However, DUBs may have a multidomain structure, and some of them associate with other proteins, including E3 ubiquitin ligases [23],[44]. We show that Notch is only able to interact with eIF3f in the presence of the E3 ubiquitin ligase DTX and that a monoubiquitinated form of Notch is probably the substrate targeted by the DUB. Therefore any reconstituted in vitro system will be difficult to set up.

As JAMM DUBs are commonly found in association with large protein complexes, on one hand, and eIF3f belongs to the eIF3 complex, on the other hand, it will be of great interest to determine whether eIF3f can work as a DUB outside of the translation complex or whether the active form of eIF3f is associated with the whole translation initiation complex. If it were the case, beside an analogy and homology in the molecular architecture of the other Zomes members (Cop9 signalosome and proteasome, [45]), eIF3 would also harbor an enzymatically-conserved organization.

### Do Translation Factors Have Multiple Functions?

Beyond acting as a translation initiation factor, eIF3f fulfills other functions, probably independently of the eIF3 complex. For instance, it was shown to inhibit HIV-1 replication [46] by modulating the sequence-specific recognition of the HIV-1 pre-mRNA by the splice factor 9G8. It was also recently proposed to serve as a scaffold in coordinating mTor and SGK1 actions on skeletal muscle growth [47] and to interact genetically and physically with TRC8, an E3 ubiquitin ligase of the RING family with several TM domains [48]. On the other hand, there are at least two different types of eIF3 complexes in the cell, localized in different subcellular fractions. One complex lacks eIF3a and eIF3f, while the other consists of eIF3a–c and eIF3f. Phosphorylated eIF3f may predominantly localize to the nucleus and join a complex containing at least b and c during apoptosis. Therefore, the nuclear eIF3 complex is likely to have functions other than translation initiation [49]. In yeast, affinity purification and LC-MS/MS was employed to characterize the eIF3 interactome, which was found to contain 230 proteins [50]. This led to the proposal that eIF3 assembles into a large supercomplex, the translasome, which contains elongation factors, tRNA synthases, 40S and 60S ribosomal proteins, chaperones, and the proteasome. On the other hand, eIF3 also associates with importins-β, a critical event for normal cell growth. These data suggest that translasomes are dynamically localized within the cell, and eIF3 could shuttle between the cytoplasm and the nucleus in a cell cycle-dependent manner. All these data are in agreement with the hypothesis that eIF3f, and maybe part of eIF3, could have multiple functions in the cells. Our results now add an enzymatic activity to these various properties; it remains to be elucidated whether this activity is necessary to fulfill all eIF3f functions.

Recent data suggest that other translation factors, many of which exist in several copies in the genome (as does eIF3f), have “part-time jobs” outside of their usual function in translation [51]. It is the case, for example, for eIF4A, which exhibits an RNA helicase activity in translation initiation and which was shown to act in a translation-independent manner as a negative regulator of Dpp/BMP signaling in drosophila [52]. eIF4A affects Mad protein level and was suggested to act as an E3 ubiquitin ligase.

In parallel also to ribosomal proteins, which have been recently shown to function outside of the ribosome and to be recruited in some cases for a function unrelated to the ribosome or its synthesis [53], we propose that eIF3f would be another example of a housekeeping protein that also plays a specific role in the Notch signaling pathway.

## Materials and Methods

### shDUB Library

shDUB library was described in [10] (see also [40],[54],[55]). Additive shRNAs are listed in Table 1. The pool targeting AMSH used as a control in Figure 7B is the number 139 of the library.

### Constructs

All myc-tagged Notch constructs (ΔE, ΔE-LLFF, FL, and NIC) were already described [18],[56] and were gifts from R. Kopan (Washington University, St. Louis, MO). These Notch constructs are all deleted from aa 2183 of murine Notch1 and fused to a hexameric myc tag at the carboxy terminus. Notch1 retroviral vector encodes a full-length human Notch1 with an HA-epitope tag inserted between EGF repeats 22 and 23 (gift of J. Aster, Harvard medical school, Boston, USA).

All WT and deletion mutants of eIF3f (containing N-terminal tags), as well as GST-fused eIF3f constructs, were gifts from S. Leibovitch (Montpellier, France), except the catalytic mutant. meIF3f Mut was generated from HA-tagged WT meIF3f by two successive site-directed mutagenesis using, respectively, the oligonucleotides 5′-GCCACAGGCGCTGCAGCCACAGAACACTCAGTGCTG-3′ and its complementary DNA, and 5′-GACATCACAGCTGCAGCCCTGCTGATCCATGAG-3′ and its complementary DNA (HDI(aa181–183) mutated in AAA and IHE(aa190–192) mutated in GIL). 6xHis-tagged Ubiquitin construct was a gift from M. Treier (European Molecular Biology Laboratory, Heidelberg, Germany).

HIS-tagged eIF3f MPN constructs were built by inserting the amino acids 95 to 226 of meIF3f WT or meIF3f Mut in pet28a vector using NdeI and BamHI restriction sites.

Retroviral meIF3f vectors were constructed first by adding a S-Tag in the N-terminus end of meIF3f WT or meIF3f mutated in the catalytic domain (HDI (aa181–183) mutated in AAA). Then S-tagged meIF3f constructions were inserted in pMSCVpuro vector using HindIII and ClaI restriction sites. The puromycin resistance gene was replaced by S-Tagged meIF3f, whose expression is by the way under the control of PGK promoter.

CSL-Luc was a gift from T. Honjo (Kyoto University, Japan) and is referred to as pGa981-6 in [30]. NDFIP2 was a gift from S. Kumar (Adelaide University, Australia) and is referred to in [19]. Both BPLF1 constructs were described in [24].

### Antibodies

We used anti-myc 9E10 and anti-VSV P5D4 antibodies. Anti-Notch IC and anti-Dll1 antibodies were described, respectively, in [57] and [31].

Other antibodies for Western blot were supplied by Abcam (polyclonal anti-S-Tag), Bethyl (polyclonal anti-eIF3a), BioLegend (polyclonal anti-eIF3f), Cell Signaling (anti-Notch V1744), Covance (monoclonal anti-HA; polyclonal anti-HA), Invitrogen (polyclonal anti-GFP), Novagen (monoclonal anti-S-Tag), and Sigma (monoclonal anti-Flag M2; polyclonal anti-Flag; monoclonal anti-β-tubulin; monoclonal anti-α-tubulin, monoclonal anti-β-Actin). Secondary antibodies for immunofluorescence were supplied by Molecular Probes (Alexa Fluor conjugates).

### Cell Lines

U20S-FL cell line was established by retroviral transduction. High titers of recombinant HA-tagged Notch FL viruses were obtained 48 h after transfection of the Plat-E ecotropic packaging cell line with retroviral expression plasmids. After retroviral transduction of the U2OS cell line, clonal populations were obtained by limiting dilution. OP9-Dll1 cell line was described in [31].

MEFs stably expressing VSV-DTX and S-Tag-meIF3f were established from the DTX-expressing MEFs [27] by retrotransduction of S-tagged meIF3f vectors.

### Immunofluorescence Assays

U2OS cells were grown on glass coverslips and were transiently transfected using FuGeneHD transfection reagent (Roche, Mannheim, Germany) for 24 h. Cells were fixed with 4% paraformaldehyde and permeabilized with PBS containing 0.2% Triton X-100 for 5 min before the incubation with appropriate antibodies. Cell preparations were mounted in Mowiol (Calbiochem, Merck Biosciences, Darmstadt, Germany) and images acquired using an AxioImager microscope with ApoTome system with a 63× magnification and AxioVision software (Carl Zeiss MicroImaging Inc., Le Pecq, France).

### Ubiquitin-Conjugates Purification

293T cells were harvested 24 h after transfection and lysed in 8 M urea, 0.1 M NaH2PO4, 10 mM Tris-Hcl (pH 8), 1% Triton X-100, and 20 mM Imidazole at room temperature. His-Ub conjugated proteins were purified on chelating Sepharose beads (Pharmacia), previously charged with Nickel. Ni-bound proteins were washed extensively with the same buffer, then with a pH 6.3 buffer, and eluted in Laemmli before Western blot analysis.

### Cell Extracts, Immunoprecipitations, and Immunoblots

293T cells were collected 24 h after transfection, washed in PBS buffer, and lysed in 50 mM Tris-HCl (pH 7,9), 400 mM NaCl, 5 mM MgCl_2_, 1% Triton X-100, supplemented with protease inhibitor cocktail (Roche). Cell extracts were cleared by centrifugation at 14,000 rpm for 20 min at 4°C. Immunoprecipitations were performed with the appropriate antibodies in the same buffer. When indicated, the immunoprecipitates were eluted by peptide competition (2 mg/mL) for 1 h at 4°C. Samples were denatured in Laemmli buffer for SDS-PAGE resolution, and immunoblots were performed as described previously [18].

### Deconjugase Activity Assays

In bacteria functional assay: Bl21 bacteria were transfected with GST-fusions or His-tagged encoding vectors together with Ub-GFP plasmid. The bacteria were selected on agar plates and cultured in LB medium containing Chloramphenicol and Ampicillin or Kanamycin. Exponential bacteria cultures (OD = 0.4) were treated with IPTG 0.5 mM for 16 h at 23°C. Bacteria were harvested, washed, resuspended in PBS containing 20 mM N*-*Ethylmaleimide, and sonicated for 30 s on ice. After centrifugation (12,000 rpm, 10 min), clear supernatants were measured for GFP fluorescence and protein concentration. Samples were analyzed by two SDS-PAGE resolutions using for each sample 100 units of fluorescence for Ub-GFP assay and 10 µg extracts for protein expression analysis.

Ub-VS assay: HeLa cells were collected 36 h after transfection, washed with PBS, and lysed in 50 mM Tris-Cl pH 7.4, 150 mM NaCl, 1 mM DTT, 1 mM EDTA, 1 mM PMSF, and 0.5% NP40. Protein concentrations were measured. 10 µg of WCE were incubated for 1 h at 37°C with 1 µg of Ub-VS functional probe (Boston Biochem) in a 30 µL final volume of labeling buffer (50 mM Tris-Cl pH 7.4, 250 mM sucrose, 5 mM MgCl_2_, and 1 mM DTT). Finally, WCE and Ub-VS-treated samples were analyzed by Western blot.

### Coculture Assay

20,000 U2OS-FL cells/cm^2^ were grown and transiently transfected using FuGeneHD transfection reagent (Roche, Mannheim, Germany). 24 h after transfection, U2OS-FL cells were cocultured with 35,000 OP9-Dll1 cells/cm^2^. 18 h later, cocultures (done in triplicates) were lysed using Passive Lysis buffer (Promega). A fraction of cell lysates were transferred to a white 96-well plate (Berthold). Firefly and Renilla luciferase activities were measured using the luminometer Centro XS (Berthold). For Western blot analysis, part of cocultures were lysed with 8 M urea, 0.1 M NaH2PO4, 10 mM Tris-Hcl (pH 8), 1% Triton X-100, and 20 mM Imidazole.

**Table 1:** List of DUBs and selected oligonucleotide sequences present in the shDUB library Part 2.

DUB Enzyme (Putative)/Sense Oligo Sequences	Ensembl Gene ID
Ataxin-3-like	ENSG00000123594
GATCCCCCATGGATGATACCGGTTTCTTCAAGAGAGAAACCGGTATCATCCATGTTTTT	
GATCCCCGCTGACCAACTCCTGCAGATTCAAGAGATCTGCAGGAGTTGGTCAGCTTTTT	
GATCCCCCCAATAGAGAAGATGAACATTCAAGAGATGTTCATCTTCTCTATTGGTTTTT	
GATCCCCCAACAAGTTCGAGAGCAATTTCAAGAGAATTGCTCTCGAACTTGTTGTTTTT	
Ataxin-3	ENSG00000066427
GATCCCCGCAGATCTCCGCAGGGCTATTCAAGAGATAGCCCTGCGGAGATCTGCTTTTT	
GATCCCCGCAAATGATGGCTCAGGAATTCAAGAGATTCCTGAGCCATCATTTGCTTTTT	
GATCCCCATCTTACTTCAGAAGAGCTTTCAAGAGAAGCTCTTCTGAAGTAAGATTTTTT	
GATCCCCCTCCTGCAGATGATTAGGGTTCAAGAGACCCTAATCATCTGCAGGAGTTTTT	
JOSD3	ENSG00000166012
GATCCCCCTCTTCTGATAGCAGCTTATTCAAGAGATAAGCTGCTATCAGAAGAGTTTTT	
GATCCCCTCATTGAAGCAAATGAATGTTCAAGAGACATTCATTTGCTTCAATGATTTTT	
GATCCCCGACCAGAAGGAAGGAGAAATTCAAGAGATTTCTCCTTCCTTCTGGTCTTTTT	
GATCCCCTTCCCTGTAAGACTGAGTGTTCAAGAGACACTCAGTCTTACAGGGAATTTTT	
HIN-1	ENSG00000164164
GATCCCCCAGAGAGAAATTTGAAGCGTTCAAGAGACGCTTCAAATTTCTCTCTGTTTTT	
GATCCCCAGTATAAAGAAAGCTCTGCTTCAAGAGAGCAGAGCTTTCTTTATACTTTTTT	
GATCCCCAAGTGCCCTTTCTCTTATGTTCAAGAGACATAAGAGAAAGGGCACTTTTTTT	
GATCCCCAAGAAAGCTCTGCTATGTGTTCAAGAGACACATAGCAGAGCTTTCTTTTTTT	
Otubain-1	ENSG00000167770
GATCCCCGACCAGGCCTGACGGCAACTTCAAGAGAGTTGCCGTCAGGCCTGGTCTTTTT	
GATCCCCGAGCAAGGAAGACCTGGTGTTCAAGAGACACCAGGTCTTCCTTGCTCTTTTT	
GATCCCCGAGATTGCTGTGCAGAACCTTCAAGAGAGGTTCTGCACAGCAATCTCTTTTT	
GATCCCCGTTCTTCGAGCACTTCATCTTCAAGAGAGATGAAGTGCTCGAAGAACTTTTT	
Otubain-2	ENSG00000089723
GATCCCCGACTTCTGCACTCACGAAGTTCAAGAGACTTCGTGAGTGCAGAAGTCTTTTT	
GATCCCCGTGGAGTACGTGGACGAGATTCAAGAGATCTCGTCCACGTACTCCACTTTTT	
GATCCCCGGATGGCTCAGTGTCCAGCTTCAAGAGAGCTGGACACTGAGCCATCCTTTTT	
GATCCCCCCGAGCAGACTTCTTCCGGTTCAAGAGACCGGAAGAAGTCTGCTCGGTTTTT	
RPN8	ENSG00000103035
GATCCCCGTCAATGCCAGGGAAAGAATTCAAGAGATTCTTTCCCTGGCATTGACTTTTT	
GATCCCCGCTTCTGGATATCAGGAGCTTCAAGAGAGCTCCTGATATCCAGAAGCTTTTT	
GATCCCCGAAAGTACTTGATGTATCGTTCAAGAGACGATACATCAAGTACTTTCTTTTT	
GATCCCCGAATGACATTGCCATCAACTTCAAGAGAGTTGATGGCAATGTCATTCTTTTT	
TL132	ENSG00000188933
GATCCCCGACACTCATATTAAGCCTATTCAAGAGATAGGCTTAATATGAGTGTCTTTTT	
GATCCCCAGATGGCAGACACAAGCAGTTCAAGAGACTGCTTGTGTCTGCCATCTTTTTT	
GATCCCCGAAGACAGCACTGATGACCTTCAAGAGAGGTCATCAGTGCTGTCTTCTTTTT	
GATCCCCCCCAAACTGCAAGTGGTACTTCAAGAGAGTACCACTTGCAGTTTGGGTTTTT	
TRABID	ENSG00000019995
GATCCCCGGAGGAACTTGAAGTAGACTTCAAGAGAGTCTACTTCAAGTTCCTCCTTTTT	
GATCCCCGTGGTTCAAGTGATGTTGGTTCAAGAGACCAACATCACTTGAACCACTTTTT	
GATCCCCGTGTATTCCAGCAATGGTGTTCAAGAGACACCATTGCTGGAATACACTTTTT	
GATCCCCGAAGACTGGGCATTTATACTTCAAGAGAGTATAAATGCCCAGTCTTCTTTTT	
USP42	ENSG00000106346
GATCCCCCCAAGAAGATGCCCATGAATTCAAGAGATTCATGGGCATCTTCTTGGTTTTT	
GATCCCCCTGTAACCTCTCTGATCGGTTCAAGAGACCGATCAGAGAGGTTACAGTTTTT	
GATCCCCGCACATATTACCCAGGCACTTCAAGAGAGTGCCTGGGTAATATGTGCTTTTT	
GATCCCCCTGGTCAGTTAATAGGTCCTTCAAGAGAGGACCTATTAACTGACCAGTTTTT	
USP48	ENSG00000090686
GATCCCCCACATTTCTTCAAGTGTGGTTCAAGAGACCACACTTGAAGAAATGTGTTTTT	
GATCCCCCAGAATGCAACAAGAAAGATTCAAGAGATCTTTCTTGTTGCATTCTGTTTTT	
GATCCCCGGCTTGTTTCTAAAGAGGCTTCAAGAGAGCCTCTTTAGAAACAAGCCTTTTT	
GATCCCCGTTCGTGGTGAGAAAGCACTTCAAGAGAGTGCTTTCTCACCACGAACTTTTT	
USP49	ENSG00000164663
GATCCCCGATTGGGGTCCATGTCGTCTTCAAGAGAGACGACATGGACCCCAATCTTTTT	
GATCCCCGCTAGAAAGCAGTTAATGATTCAAGAGATCATTAACTGCTTTCTAGCTTTTT	
GATCCCCGCTAGAAAGCAGTTAATGATTCAAGAGATCATTAACTGCTTTCTAGCTTTTT	
GATCCCCCAGGACGCGCAGGAATTTCTTCAAGAGAGAAATTCCTGCGCGTCCTGTTTTT	
USP54	ENSG00000166348
GATCCCCCCAGCATATTGGGACCAGATTCAAGAGATCTGGTCCCAATATGCTGGTTTTT	
GATCCCCCAGCAGAAATGGAGCATGGTTCAAGAGACCATGCTCCATTTCTGCTGTTTTT	
GATCCCCCTAACCTACACTGCCACACTTCAAGAGAGTGTGGCAGTGTAGGTTAGTTTTT	
GATCCCCGGTTTACCTAAAGCACCAGTTCAAGAGACTGGTGCTTTAGGTAAACCTTTTT	
VCIP135	ENSG00000175073
GATCCCCGCTCTGTTCCATGACTTCATTCAAGAGATGAAGTCATGGAACAGAGCTTTTT	
GATCCCCCAAACCAATCTGTATTGCATTCAAGAGATGCAATACAGATTGGTTTGTTTTT	
GATCCCCGCAGTAATGGATAATCGCCTTCAAGAGAGGCGATTATCCATTACTGCTTTTT	
GATCCCCGCATAATACAGGGACAGACTTCAAGAGAGTCTGTCCCTGTATTATGCTTTTT	
OTUD2, YOD1	ENSG00000180667
GATCCCCCCAGGCCCAGAAGTTCACCTTCAAGAGAGGTGAACTTCTGGGCCTGGTTTTT	
GATCCCCCGTGGTGCTTCTAGTTACGTTCAAGAGACGTAACTAGAAGCACCACGTTTTT	
GATCCCCGCGATCCAGACTTCTATAGTTCAAGAGACTATAGAAGTCTGGATCGCTTTTT	
GATCCCCGCACTGGAATTAGCAGATGTTCAAGAGACATCTGCTAATTCCAGTGCTTTTT	
A20	ENSG00000118503
GATCCCCCAAACTCCCAAAGCTGAACTTCAAGAGAGTTCAGCTTTGGGAGTTTGTTTTT	
GATCCCCGTTTGGAATCAGGTTCCAATTCAAGAGATTGGAACCTGATTCCAAACTTTTT	
GATCCCCGAAATACACATATTTGTCCTTCAAGAGAGGACAAATATGTGTATTTCTTTTT	
GATCCCCGGAAACAGACACACGCAACTTCAAGAGAGTTGCGTGTGTCTGTTTCCTTTTT	
BRCC36, BRCC3	ENSG00000185515
GATCCCCCTGAAATGCGCACAGTTGCTTCAAGAGAGCAACTGTGCGCATTTCAGTTTTT	
GATCCCCGTGTGCCTTGAATCAGCAGTTCAAGAGACTGCTGATTCAAGGCACACTTTTT	
GATCCCCGATCCATAATGGCTCAGTGTTCAAGAGACACTGAGCCATTATGGATCTTTTT	
GATCCCCCCAACAGCATTTGCAGGAATTCAAGAGATTCCTGCAAATGCTGTTGGTTTTT	
Cezanne-1	ENSG00000163113
GATCCCCGGGAGTTGAGAAGGAAGCGTTCAAGAGACGCTTCCTTCTCAACTCCCTTTTT	
GATCCCCGAGTTTCACGTCTTTGTCCTTCAAGAGAGGACAAAGACGTGAAACTCTTTTT	
GATCCCCCCCTGAAGATGGGTCACCGTTCAAGAGACGGTGACCCATCTTCAGGGTTTTT	
GATCCCCGGACAAGAAGAGAGCAGATTTCAAGAGAATCTGCTCTCTTCTTGTCCTTTTT	
Cezanne-2	ENSG00000169918
GATCCCCGCCCATTTCTCTGCCCTTGTTCAAGAGACAAGGGCAGAGAAATGGGCTTTTT	
GATCCCCGCTGAACCTTCTGCACAGCTTCAAGAGAGCTGTGCAGAAGGTTCAGCTTTTT	
GATCCCCCAATGTTAAGAGACTCAGGTTCAAGAGACCTGAGTCTCTTAACATTGTTTTT	
GATCCCCCGAGCTGTAAACGGCTTCTTTCAAGAGAAGAAGCCGTTTACAGCTCGTTTTT	
CSN5, JAB1, SGN5	ENSG00000121022
GATCCCCCCATGATCATTATGGACAGTTCAAGAGACTGTCCATAATGATCATGGTTTTT	
GATCCCCGGATCACCATTACTTTAAGTTCAAGAGACTTAAAGTAATGGTGATCCTTTTT	
GATCCCCCCCGAGTAAATGCTCAGGCTTCAAGAGAGCCTGAGCATTTACTCGGGTTTTT	
GATCCCCCTACCATAGAAGCTATCCATTCAAGAGATGGATAGCTTCTATGGTAGTTTTT	
CSN6, SGN6	ENSG00000168090
GATCCCCGTTGAACCCTATGACCAAGTTCAAGAGACTTGGTCATAGGGTTCAACTTTTT	
GATCCCCGATTATCATTGACAAGGAATTCAAGAGATTCCTTGTCAATGATAATCTTTTT	
GATCCCCCATCTCAGACCACTGGATCTTCAAGAGAGATCCAGTGGTCTGAGATGTTTTT	
GATCCCCCGCATTGGTGTAGACCACGTTCAAGAGACGTGGTCTACACCAATGCGTTTTT	
DUB-3	ENSG00000182945
GATCCCCCTACATGCTGTCCCGGGAGTTCAAGAGACTCCCGGGACAGCATGTAGTTTTT	
GATCCCCGCAGGAAGATGCCCATGAATTCAAGAGATTCATGGGCATCTTCCTGCTTTTT	
GATCCCCGGACACCACCCTCATCCACTTCAAGAGAGTGGATGAGGGTGGTGTCCTTTTT	
GATCCCCCAAGCAGGTAGATCATCACTTCAAGAGAGTGATGATCTACCTGCTTGTTTTT	
eIF-epsilon	ENSG00000175390
GATCCCCGTGGCTGTTGACATGGAATTTCAAGAGAATTCCATGTCAACAGCCACTTTTT	
GATCCCCGCCTACGTCAGCACTTTAATTCAAGAGATTAAAGTGCTGACGTAGGCTTTTT	
GATCCCCCGCATCGGAGTTGACCTGATTCAAGAGATCAGGTCAACTCCGATGCGTTTTT	
GATCCCCGATGAAGTGGCTGTTGACATTCAAGAGATGTCAACAGCCACTTCATCTTTTT	
eIF-3-gamma	ENSG00000147677
GATCCCCGAAGGACAAGGAACTGAAGTTCAAGAGACTTCAGTTCCTTGTCCTTCTTTTT	
GATCCCCGTGCAGATAGATGGCCTTGTTCAAGAGACAAGGCCATCTATCTGCACTTTTT	
GATCCCCGTCCAATATCAGATGGAAATTCAAGAGATTTCCATCTGATATTGGACTTTTT	
GATCCCCCTTGAAAAGAAGTCAGCTGTTCAAGAGACAGCTGACTTCTTTTCAAGTTTTT	
ENSG00000197767	ENSG00000197767
GATCCCCGATTGCCAAGAATGTGCAATTCAAGAGATTGCACATTCTTGGCAATCTTTTT	
GATCCCCGCTCCCTGCTAAACCTCTCTTCAAGAGAGAGAGGTTTAGCAGGGAGCTTTTT	
GATCCCCGATTGCCAAGAATGTGCAATTCAAGAGATTGCACATTCTTGGCAATCTTTTT	
GATCCCCGCAGGAAGATGCCCATGAATTCAAGAGATTCATGGGCATCTTCCTGCTTTTT	
ENSG00000198817	ENSG00000198817
GATCCCCGCCTATGTCAGCACTTTAATTCAAGAGATTAAAGTGCTGACATAGGCTTTTT	
GATCCCCGATGAAGTGGCTGTTGACATTCAAGAGATGTCAACAGCCACTTCATCTTTTT	
GATCCCCCGCATCGGAGTTGAGCTGATTCAAGAGATCAGCTCAACTCCGATGCGTTTTT	
GATCCCCGAGTGATTGGACTCTTAAGTTCAAGAGACTTAAGAGTCCAATCACTCTTTTT	
HIN-1-like	ENSG00000118976
GATCCCCCTAATCTGGAACCTAATGTTTCAAGAGAACATTAGGTTCCAGATTAGTTTTT	
GATCCCCGTTGGAGACAAATGTCAAGTTCAAGAGACTTGACATTTGTCTCCAACTTTTT	
GATCCCCCTGCTGTTGCTGCTGCTGATTCAAGAGATCAGCAGCAGCAACAGCAGTTTTT	
GATCCCCGACAACGTGAGTTCTCTAGTTCAAGAGACTAGAGAACTCACGTTGTCTTTTT	
HIN-6	ENSG00000189401
GATCCCCGACCAGCTGGTGTTCAGCGTTCAAGAGACGCTGAACACCAGCTGGTCTTTTT	
GATCCCCGACCTGGCCAAGATGAATCTTCAAGAGAGATTCATCTTGGCCAGGTCTTTTT	
GATCCCCCGACGACTTCATGATCTACTTCAAGAGAGTAGATCATGAAGTCGTCGTTTTT	
GATCCCCGAAGCCGATCATCCTGGTCTTCAAGAGAGACCAGGATGATCGGCTTCTTTTT	
IFP38	ENSG00000187684
GATCCCCGCCTATGTCAGCACTTTAATTCAAGAGATTAAAGTGCTGACATAGGCTTTTT	
GATCCCCGTTTCTCCAAATGAGCTCATTCAAGAGATGAGCTCATTTGGAGAAACTTTTT	
GATCCCCCGGCCATATGAGCATCAAATTCAAGAGATTTGATGCTCATATGGCCGTTTTT	
GATCCCCGTGTTCACACCTTTGACAGTTCAAGAGACTGTCAAAGGTGTGAACACTTTTT	
*JAMM2*	ENSG00000162601
GATCCCCCAGAGAATGGCCTTATTCCTTCAAGAGAGGAATAAGGCCATTCTCTGTTTTT	
GATCCCCGAATTCTAGCAGTGATCTCTTCAAGAGAGAGATCACTGCTAGAATTCTTTTT	
GATCCCCCTGGTGTGATGCAAAGGACTTCAAGAGAGTCCTTTGCATCACACCAGTTTTT	
GATCCCCCAGAGAATGGCCTTATTCCTTCAAGAGAGGAATAAGGCCATTCTCTGTTTTT	
*JAMM3*	ENSG00000008382
GATCCCCGTAACATCCTTTGCAGCCATTCAAGAGATGGCTGCAAAGGATGTTACTTTTT	
GATCCCCGAGGAGATCTACCAGAGCCTTCAAGAGAGGCTCTGGTAGATCTCCTCTTTTT	
GATCCCCCATCGACGCACAGATGGACTTCAAGAGAGTCCATCTGTGCGTCGATGTTTTT	
GATCCCCCACCCTGGTGGAAGTAACATTCAAGAGATGTTACTTCCACCAGGGTGTTTTT	
*JOSD1*	ENSG00000100221
GATCCCCATGGCAACTACGATGTGAATTCAAGAGATTCACATCGTAGTTGCCATTTTTT	
GATCCCCAGGCTATGAAGCTGTTTGGTTCAAGAGACCAAACAGCTTCATAGCCTTTTTT	
GATCCCCCTCAAGATGCCCGAGTGGATTCAAGAGATCCACTCGGGCATCTTGAGTTTTT	
GATCCCCACGTCTTCCAGGACAGCAATTCAAGAGATTGCTGTCCTGGAAGACGTTTTTT	
*JOSD2*	ENSG00000161677
GATCCCCCCGGCAACTATGATGTCAATTCAAGAGATTGACATCATAGTTGCCGGTTTTT	
GATCCCCCGTTCTGCAGCAGCAGCTCTTCAAGAGAGAGCTGCTGCTGCAGAACGTTTTT	
GATCCCCCTATGATGTCAATGTGATCTTCAAGAGAGATCACATTGACATCATAGTTTTT	
GATCCCCCCTGGACTCCAAGCTGCGGTTCAAGAGACCGCAGCTTGGAGTCCAGGTTTTT	
OTUD1	ENSG00000165312
GATCCCCCTTCCGACTGAGCGAGCACTTCAAGAGAGTGCTCGCTCAGTCGGAAGTTTTT	
GATCCCCGATGCCCGCCTTCTCCTCCTTCAAGAGAGGAGGAGAAGGCGGGCATCTTTTT	
GATCCCCGCAGGACAAGTATCTGCGGTTCAAGAGACCGCAGATACTTGTCCTGCTTTTT	
GATCCCCGCCGGTGATCGTCTCCAGGTTCAAGAGACCTGGAGACGATCACCGGCTTTTT	
OTUD5	ENSG00000068308
GATCCCCCAGGAAGCGGAAAAACAATTTCAAGAGAATTGTTTTTCCGCTTCCTGTTTTT	
GATCCCCTGCCGACTACTTCTCCAACTTCAAGAGAGTTGGAGAAGTAGTCGGCATTTTT	
GATCCCCGGAGGATGGCGCCTGTCTCTTCAAGAGAGAGACAGGCGCCATCCTCCTTTTT	
GATCCCCCAGGAATACCTAGACAGTATTCAAGAGATACTGTCTAGGTATTCCTGTTTTT	
OTUD6B	ENSG00000155100
GATCCCCCGGATAGCTGAAGCTGAAATTCAAGAGATTTCAGCTTCAGCTATCCGTTTTT	
GATCCCCTAGAGATAATACAGGCAGATTCAAGAGATCTGCCTGTATTATCTCTATTTTT	
GATCCCCCAAACCCTAATACAGGAGATTCAAGAGATCTCCTGTATTAGGGTTTGTTTTT	
GATCCCCCCACTAATACTTGTATATATTCAAGAGATATATACAAGTATTAGTGGTTTTT	
POH1, RPN11	ENSG00000115233
GATCCCCCAAGTCTATATCTCTTCCCTTCAAGAGAGGGAAGAGATATAGACTTGTTTTT	
GATCCCCCAAGCCATCTATCCAGGCATTCAAGAGATGCCTGGATAGATGGCTTGTTTTT	
GATCCCCGGCCGGAGATGGTTGTTGGTTCAAGAGACCAACAACCATCTCCGGCCTTTTT	
GATCCCCCAGCTGGCAATAAAGAATGTTCAAGAGACATTCTTTATTGCCAGCTGTTTTT	
PRP8	ENSG00000174231
GATCCCCGGACAACCCCAACCTGCTGTTCAAGAGACAGCAGGTTGGGGTTGTCCTTTTT	
GATCCCCCCAGATTCCCAATCGTAGATTCAAGAGATCTACGATTGGGAATCTGGTTTTT	
GATCCCCGATGTATGAGAAGATCGACTTCAAGAGAGTCGATCTTCTCATACATCTTTTT	
GATCCCCGATTCATGGGATCGTGGCATTCAAGAGATGCCACGATCCCATGAATCTTTTT	
STAMBP, AMSH	ENSG00000124356
GATCCCCGAAGAAGGAAGCAGAGGAATTCAAGAGATTCCTCTGCTTCCTTCTTCTTTTT	
GATCCCCCAGTCTCATCCATACAGCCTTCAAGAGAGGCTGTATGGATGAGACTGTTTTT	
GATCCCCGTTCCAGGAAACTGGATTCTTCAAGAGAGAATCCAGTTTCCTGGAACTTTTT	
GATCCCCGGCAGAGCTGTTAAAACGATTCAAGAGATCGTTTTAACAGCTCTGCCTTTTT	
AMSH-like	ENSG00000138134
GATCCCCCTCATGTTGCCAGAGGCCATTCAAGAGATGGCCTCTGGCAACATGAGTTTTT	
GATCCCCGAAACTGAAGGAGATTGCATTCAAGAGATGCAATCTCCTTCAGTTTCTTTTT	
GATCCCCCAATTCCTTGCTGAATGTATTCAAGAGATACATTCAGCAAGGAATTGTTTTT	
GATCCCCGCATAAAGACACTGGCATCTTCAAGAGAGATGCCAGTGTCTTTATGCTTTTT	
TL132-like	ENSG00000189423
GATCCCCGACACTCATATTAAGCCTATTCAAGAGATAGGCTTAATATGAGTGTCTTTTT	
GATCCCCCCCAAACTGCAAGTGGTACTTCAAGAGAGTACCACTTGCAGTTTGGGTTTTT	
GATCCCCGATGGCAGACACAAGCAGGTTCAAGAGACCTGCTTGTGTCTGCCATCTTTTT	
GATCCCCGTGAAGAAGACAGCACTGATTCAAGAGATCAGTGCTGTCTTCTTCACTTTTT	

## Supporting Information

Figure S1 **shRNAs affecting eIF3f level inhibit translation**. U2OS cells were transfected with ΔE-encoding vector together with three increasing doses of sh eIF3f P1. After 24 h, immunostaining was performed with anti-myc antibody, and nuclei were stained with Hoechst. The images were acquired using an Axio Imager microscope with a 20× magnification objective. The percentage of myc-positive cells was calculated in each case after counting 163, 226, 155, and 214 cells, respectively. This representative of several experiments shows that eIF3f inhibition affects the translation efficiency of ΔE-encoding vector. The same type of result was obtained with all isolated shRNAs targeting eIF3f at different doses. Note also that at the lower dose (Dose 1 in the second lane), the subcellular localization of myc staining was affected compared to ΔE alone (first lane), Notch being partially retained in extra-nuclear structures.
(0.36 MB DOC)

Figure S2 **sh eIF3f #3 does not affect Ndfip2 ubiquitination.** U2OS cells were transfected with vectors encoding GFP-tagged Ndfip2 and 6xHis-tagged Ubiquitin. We added shRNA #3 in the presence or not of the murine shRNA #3 insensitive meIF3f. Proteins were extracted in denaturing conditions and ubiquitinated products were purified on Nickel-charged beads. Whole cell extracts (WCE) and ubiquitinated products (Nickel) were analyzed by Western blot using the anti-GFP antibody to quantify the levels of ubiquitinated Ndfip2. These data suggest that neither sh eIF3f #3 nor meIF3f overexpression has an effect on Ndfip2 ubiquitination.
(0.96 MB PPT)

Figure S3 **eIF3f does interact with DTX but not with Itch/AIP4.** HEK-293T cells were transfected with vectors encoding HA-tagged meIF3f and VSV-tagged DTX or Flag-tagged Itch/AIP4. 24 h after trasnsfection, cells were lysed and proteins were extracted. meIF3f was immunoprecipitated using anti-HA antibody. Finally, whole cell extracts (WCE) and immunoprecipitates were analyzed by Western blot using the indicated antibodies. These data show that DTX is co-immunoprecipitated with eIF3f (Lane B), whereas Itch/AIP4 is not (Lane C).
(0.99 MB PPT)

Figure S4 **A chimera between ubiquitin and Notch IC is partially located outside of the nucleus and presents a transcriptional activity defect.** (A) U2OS cells were transfected with vectors encoding NIC (first lane), NIC(KR), where the Lysine 1749 of murine Notch1 was mutated to Arginine [8], or UBIC (obtained by inserting a PCR-amplified NIC(KR) at the AgeI site of UbG76V, K29/48R-GFP [28], therefore NIC being in frame 3′ to the ubiquitin sequence and the GFP being off-frame). After 24 h, immunostaining was performed with rabbit anti-Notch-IC antibody [57]. Nuclei were stained with Hoechst. The percentage of cells presenting extra-nuclear staining was calculated after counting more than 100 random transfected cells on three independent experiments. It was 4% for both NIC and NIC(KR) on average, and 18% for UBIC. These data suggest that monoubiquitination is sufficient to partially hinder NIC nuclear import. (B) U2OS cells were transfected with increasing doses of NIC (A to C), NIC(KR) (D to F), or UBIC (G to I) together with a CSL-Luciferase reporter and a pRL-TK vector encoding Renilla luciferase used as an internal control. After 24 h, relative luciferase activity was measured and cell extracts were analyzed by Western blot. CSL activation was repressed in a dose-dependent manner with UBIC, respectively, reaching 66%, 79%, and 80% of reduction compared to the similar doses of NIC.
(4.97 MB PPT)

Figure S5 **shRNAs targeting eIF3f have no effect on NIC-mediated transcriptional activation.** U2OS cells were transfected with vectors encoding CSL-firefly luciferase (Notch reporter) and TK-renilla luciferase (internal control reporter), together with NIC (Lane A) and increasing doses of eIF3f shRNA P2 (B–D), shRNA #1 (E–G), or a control pool targeting AMSH (H–J). 24 h after transfection, relative luciferase activity was measured and cell extracts were analyzed by Western blot. These data show that NIC-mediated CSL activation is not affected by shRNAs targeting eIF3f. This suggests that CSL-activation decrease observed in coculture experiments in the presence of eIF3f shRNAs is not due to an effect of these shRNAs on Notch-associated transcription factors.
(0.54 MB PPT)

## Abbreviations

CSN5: COP9 Signalosome subunit 5
Dll1: Delta-like1
DTX: Deltex1
DUB: deubiquitinating enzyme
eIF3f: eukaryotic translation initiation factor 3 subunit f
FL: full-length
JAMM: Jab/MPN domain-associated metallopeptidase
meIF3f: murine form of eIF3f
MPN: MPR1-PAD1-N-terminal
NIC: intracellular portion of Notch
PCI: Proteasome-COP9 signalosome-initiation factor 3
PIC: preinitiation complex
Ub-GFP: GFP fused to ubiquitin
Ub-VS: Ubiquitin Vinyl Sulfone
