# Supplementary figures and images for: The Translation Initiation Factor 3f (eIF3f) Exhibits a Deubiquitinase Activity Regulating Notch Activation
Source: PLoS Biol. 2010 Nov 23;8(11):e1000545. doi: 10.1371/journal.pbio.1000545 (PMC2990700; doi:10.1371/journal.pbio.1000545)

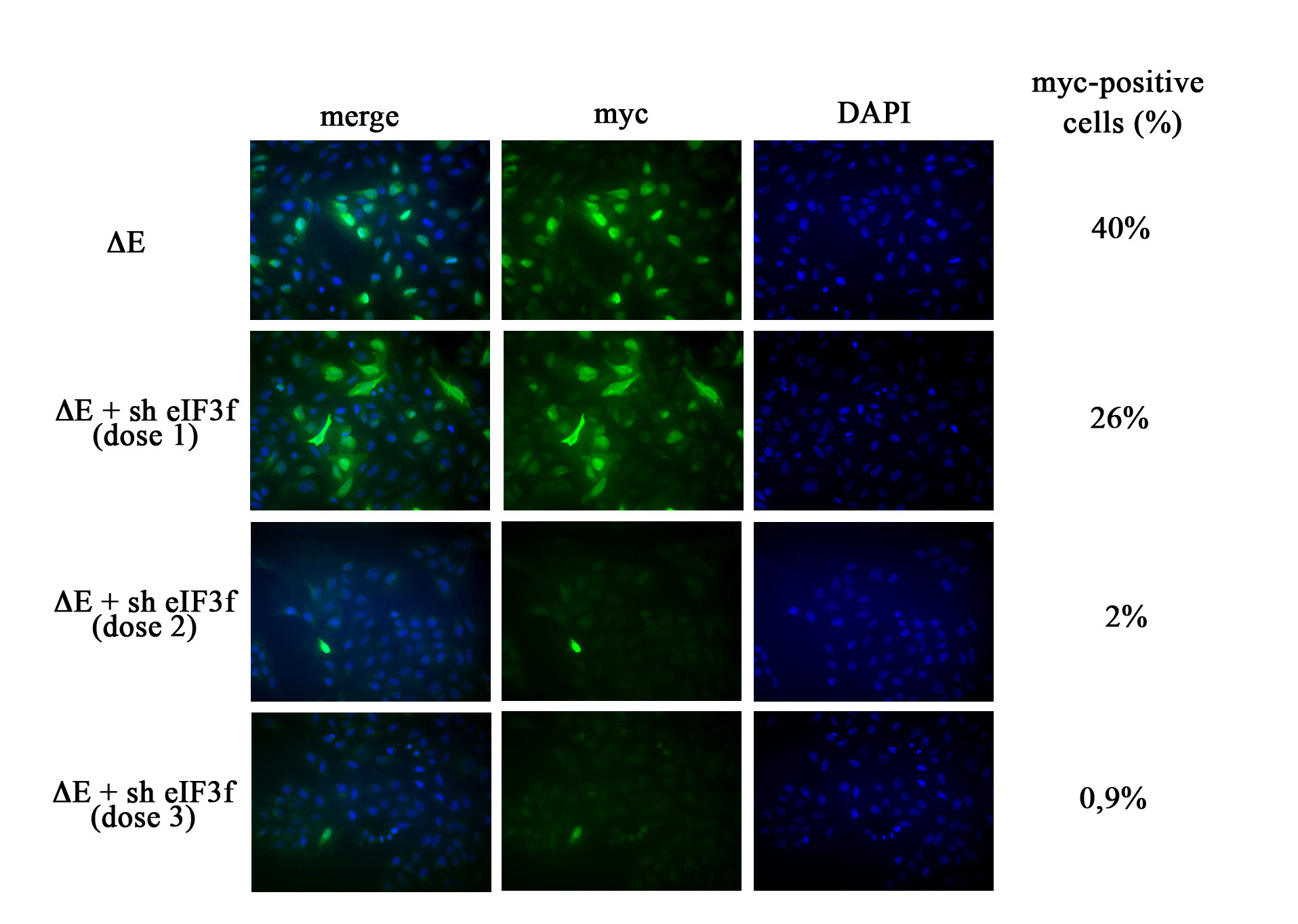

Supplement: Figure S1 — shRNAs affecting eIF3f level inhibit translation. U2OS cells were transfected with ΔE-encoding vector together with three increasing doses of sh eIF3f P1. After 24 h, immunostaining was performed with anti-myc antibody, and nuclei were stained with Hoechst. The images were acquired using an Axio Imager microscope with a 20× magnification objective. The percentage of myc-positive cells was calculated in each case after counting 163, 226, 155, and 214 cells, respectively. This representative of several experiments shows that eIF3f inhibition affects the translation efficiency of ΔE-encoding vector. The same type of result was obtained with all isolated shRNAs targeting eIF3f at different doses. Note also that at the lower dose (Dose 1 in the second lane), the subcellular localization of myc staining was affected compared to ΔE alone (first lane), Notch being partially retained in extra-nuclear structures. (0.36 MB DOC) [file pbio.1000545.s001.doc]

## Slide 1
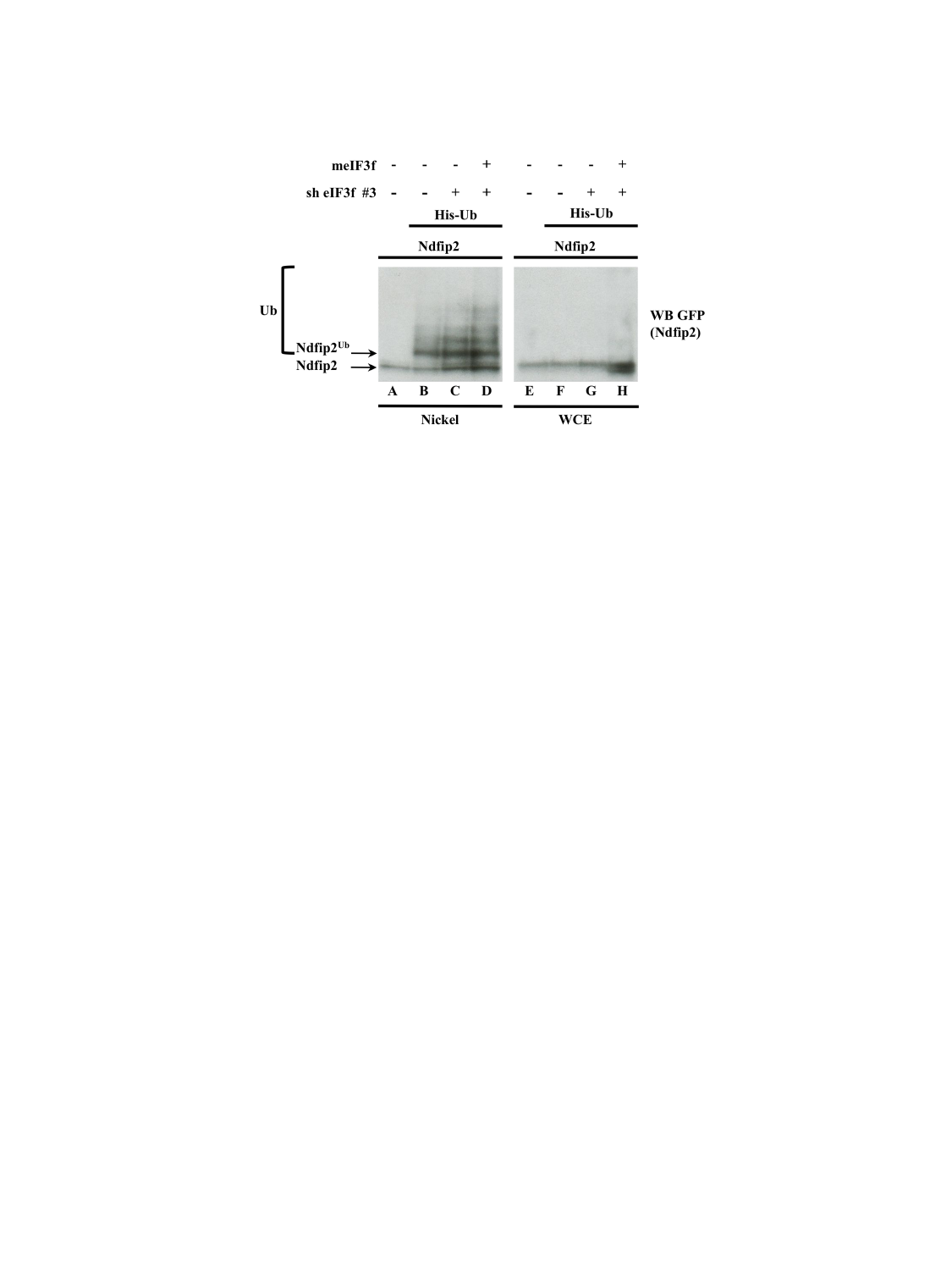

Supplement: Figure S2 — sh eIF3f #3 does not affect Ndfip2 ubiquitination. U2OS cells were transfected with vectors encoding GFP-tagged Ndfip2 and 6xHis-tagged Ubiquitin. We added shRNA #3 in the presence or not of the murine shRNA #3 insensitive meIF3f. Proteins were extracted in denaturing conditions and ubiquitinated products were purified on Nickel-charged beads. Whole cell extracts (WCE) and ubiquitinated products (Nickel) were analyzed by Western blot using the anti-GFP antibody to quantify the levels of ubiquitinated Ndfip2. These data suggest that neither sh eIF3f #3 nor meIF3f overexpression has an effect on Ndfip2 ubiquitination. (0.96 MB PPT) [file pbio.1000545.s002.ppt]

## Slide 1
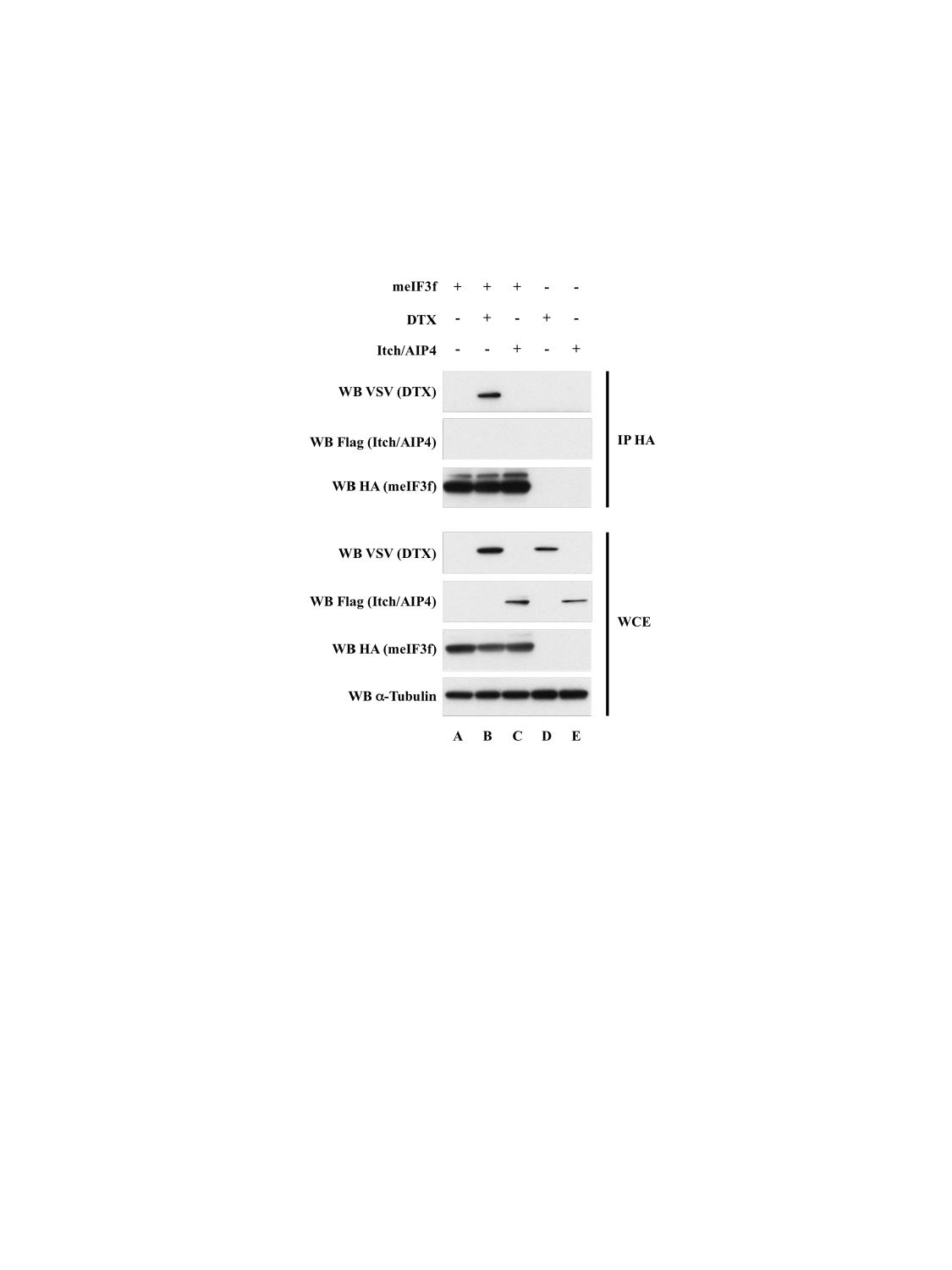

Supplement: Figure S3 — eIF3f does interact with DTX but not with Itch/AIP4. HEK-293T cells were transfected with vectors encoding HA-tagged meIF3f and VSV-tagged DTX or Flag-tagged Itch/AIP4. 24 h after trasnsfection, cells were lysed and proteins were extracted. meIF3f was immunoprecipitated using anti-HA antibody. Finally, whole cell extracts (WCE) and immunoprecipitates were analyzed by Western blot using the indicated antibodies. These data show that DTX is co-immunoprecipitated with eIF3f (Lane B), whereas Itch/AIP4 is not (Lane C). (0.99 MB PPT) [file pbio.1000545.s003.ppt]

## Slide 1
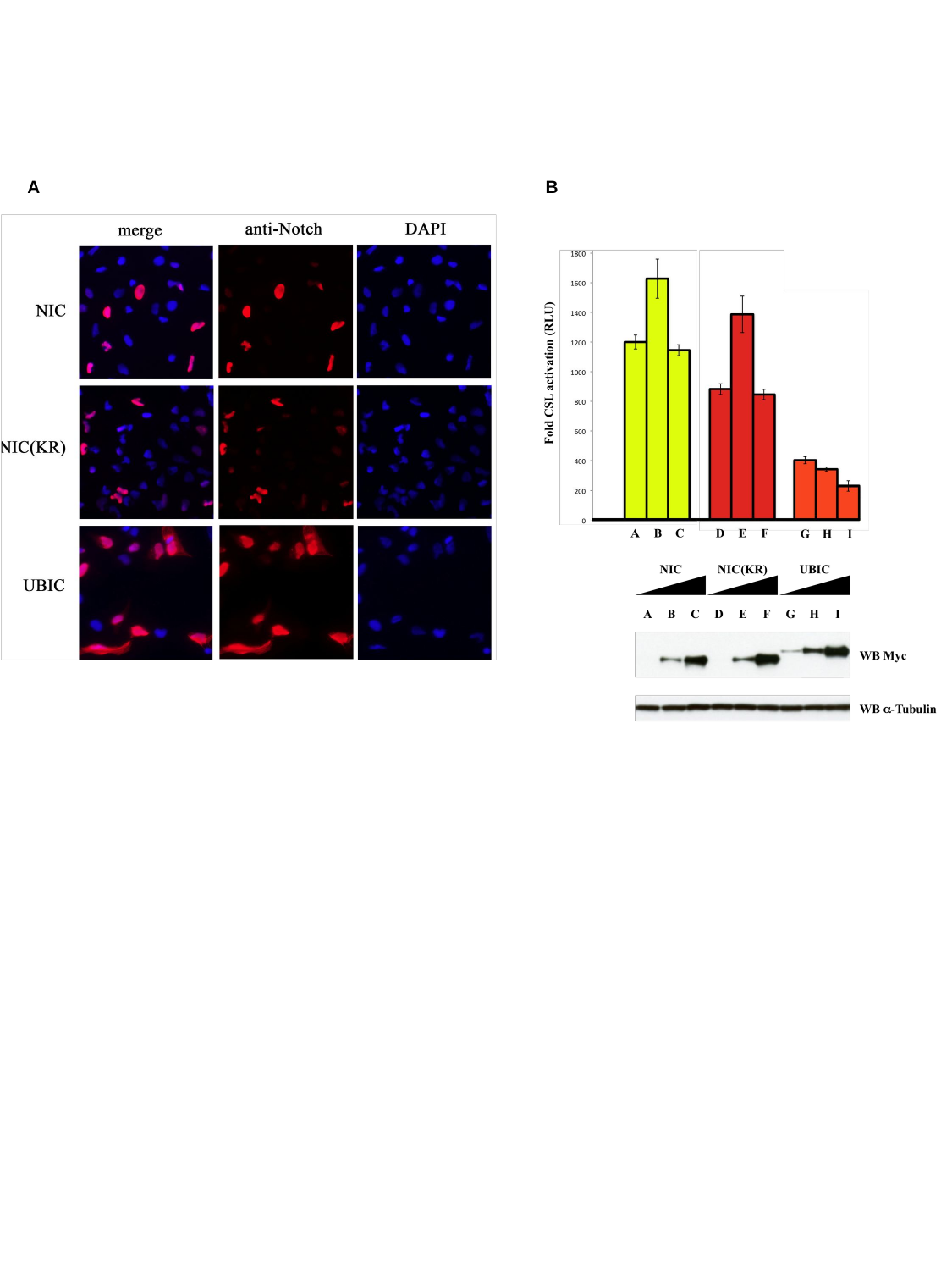

A
B

Supplement: Figure S4 — A chimera between ubiquitin and Notch IC is partially located outside of the nucleus and presents a transcriptional activity defect. (A) U2OS cells were transfected with vectors encoding NIC (first lane), NIC(KR), where the Lysine 1749 of murine Notch1 was mutated to Arginine [8], or UBIC (obtained by inserting a PCR-amplified NIC(KR) at the AgeI site of UbG76V, K29/48R-GFP [28], therefore NIC being in frame 3′ to the ubiquitin sequence and the GFP being off-frame). After 24 h, immunostaining was performed with rabbit anti-Notch-IC antibody [57]. Nuclei were stained with Hoechst. The percentage of cells presenting extra-nuclear staining was calculated after counting more than 100 random transfected cells on three independent experiments. It was 4% for both NIC and NIC(KR) on average, and 18% for UBIC. These data suggest that monoubiquitination is sufficient to partially hinder NIC nuclear import. (B) U2OS cells were transfected with increasing doses of NIC (A to C), NIC(KR) (D to F), or UBIC (G to I) together with a CSL-Luciferase reporter and a pRL-TK vector encoding Renilla luciferase used as an internal control. After 24 h, relative luciferase activity was measured and cell extracts were analyzed by Western blot. CSL activation was repressed in a dose-dependent manner with UBIC, respectively, reaching 66%, 79%, and 80% of reduction compared to the similar doses of NIC. (4.97 MB PPT) [file pbio.1000545.s004.ppt]

## Slide 1
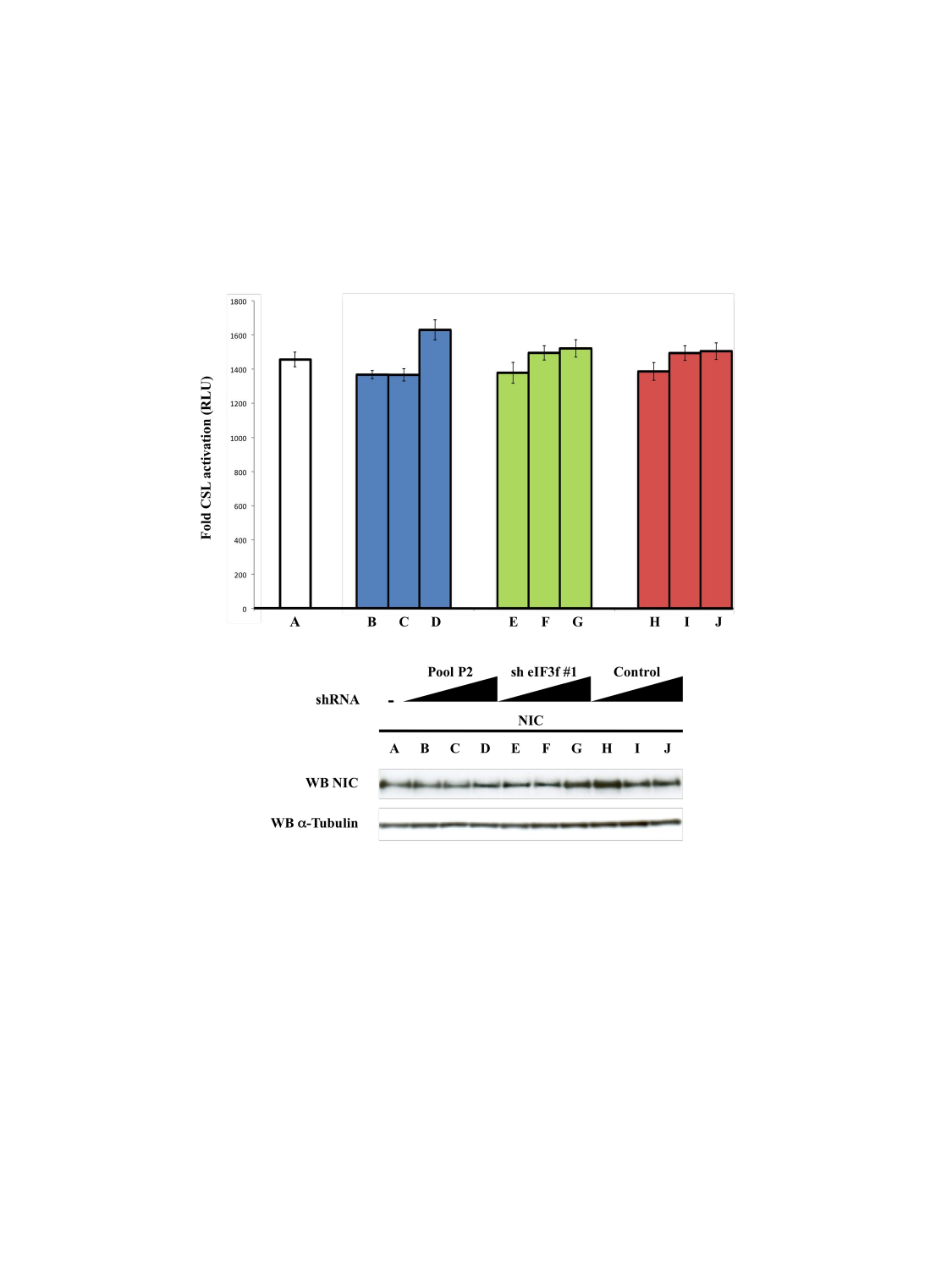

Supplement: Figure S5 — shRNAs targeting eIF3f have no effect on NIC-mediated transcriptional activation. U2OS cells were transfected with vectors encoding CSL-firefly luciferase (Notch reporter) and TK-renilla luciferase (internal control reporter), together with NIC (Lane A) and increasing doses of eIF3f shRNA P2 (B–D), shRNA #1 (E–G), or a control pool targeting AMSH (H–J). 24 h after transfection, relative luciferase activity was measured and cell extracts were analyzed by Western blot. These data show that NIC-mediated CSL activation is not affected by shRNAs targeting eIF3f. This suggests that CSL-activation decrease observed in coculture experiments in the presence of eIF3f shRNAs is not due to an effect of these shRNAs on Notch-associated transcription factors. (0.54 MB PPT) [file pbio.1000545.s005.ppt]
